# Experimental evaluation of differential voltage protection scheme based on a coherence function applied to AC machine stator windings

**DOI:** 10.1038/s41598-025-93210-2

**Published:** 2025-04-11

**Authors:** R. A. Mahmoud

**Affiliations:** Department of Electrical Power and Machines Engineering (PME), College of Engineering Science and Technology, Misr University for Science and Technology (MUST), 6th of October City, Giza Egypt

**Keywords:** Induction machines, Shunt faults, Turn-to-turn faults, Fault detection and discrimination, Voltage transformers, Differential voltage relays, Coherence measure, Energy science and technology, Engineering

## Abstract

A coherence algorithm is used to detect and classify electrical faults in the stator windings of the induction machine in this paper. The approach can definitely define turn-to-turn and internal shunt faults that occur on the three-phase machine windings. It can also distinguish between the diverse types of the internal shunt faults**.** The approach requires the acquisition of three-phase voltage measurements at the midpoints and complete terminals of the stator windings of the induction machine**.** The function of the numerical differential voltage relay can be performed using cross-coherence and auto-coherence coefficients quantified for the voltage measurements to determine and categorize the various faults. A modified configuration for the three-phase stator windings of an induction motor is used to test the technique; in which, each stator winding of the equipment is re-wounded to obtain 20 taps per phase. This configuration aims to construct voltage transformers at the midpoints and complete terminals for the machine stator windings, and to facilitate making comprehensive tests to ensure the effectiveness of the coherence technique. The testing results demonstrate the effectiveness and efficiency of the advanced protection. The experimental findings indicate that the protection’s dependability and security percentages are greater than 98.0%, and the protection’s reliability and accuracy rates are nearly 97.0%. The coherence measure has the ability to discover winding faults, particularly those occurring from turn to turn, differentiate between fault locations, and categorize shunt faults located within the machine protection zone. Additionally, the proposed approach has a high-speed response that is adjustable, and new tripping curves are created.

## Introduction

Electrical machines remain an integral part of power grids. Because of their multiple applications, the machines may be susceptible to a variety of faults that need to be diagnosed and repaired^1^. It is imperative to create and develop protection and measurement techniques that facilitate the fast identification and precise evaluation of diverse machine problems within the industry. The establishment of practical and microprocessor-driven systems/processes for fault evaluation and the development of extensive methods permit users to adopt the most appropriate approach for fault diagnosis and analysis^2^. Most faults are dedicated to the stator windings of the AC machines. Winding faults are common due to high temperatures, short-circuit currents, mechanical stresses, magnetic forces and insulation breakdown^3,4^. In general, fault diagnosis methods for the machine stator windings use magnetic flux, voltage, current, reactive power, or a combination of them. Despite being more serious than the turn-to‐turn winding faults, the line‐to‐line and line‐to‐ground faults can be identified easier by protective relays within a fraction of a second^5^. It is difficult to detect the turn-to‐turn fault at the initial stage because it does not remarkably affect the two terminal winding currents. Thus, it is imperative to monitor inter-turn faults in order to diagnose their onset and avert any machine failure. The insulation breakdown is not serious at the first stage, however, if it is not identified early, it may result in a dangerous fault^6^. Actually, a high‐induced current, which originates due to the inter-turn fault in the winding, circulates in the shorted turns and may lead to winding deterioration and machine malfunction^7^. Early fault detection can reduce a machine’s degradation, which can be maintained by rewinding its defective winding and putting it back into service^8^. Numerous researches were presented to diagnose the faults in AC machine stator windings.

In^9^, a slope of a Lissajous curve was measured to ascertain the inter-turn faults in turbo‐generators. In^10,11^, the magnetic flux sensors were used to figure out Turn-to-Turn Fault (TTF) in induction machine stator windings. These sensors possess the capability to measure the air-gap and stray magnetic fields with significant sensitivity. In^12^, Convolutional Neural Networks (CNNs) and current signature analysis were used to diagnose machine faults. The CNNs algorithm was used to find different faults in machine windings, which automatically removed relevant features from the current measurements. In^13^, a combination of machine learning and wavelet transform was used to figure out and diagnose faults. The technique of wavelet transform was harnessed for features extraction from machine output, while the technique of machine learning was exploited for faults classification, which increased the accuracy of the protection. In^14^, a fault diagnosis was accomplished using a combination of transfer learning and few-shot learning to protect asynchronous machines. In^15^, the effectiveness and robustness of the approach were increased by integrating the outputs of multiple deep learning (DL) models for machine faults diagnosis. In^16^, the precision of faults detection and classification was enhanced by the integration of machine learning with wavelet packet decomposition by extracting detailed frequency information. In^17^, edge computing was applied to identify faults online for induction machines. Low-latency fault detection was achieved by applying machine learning models to edge devices. In^18^, a Convolutional Neural Networks (CNNs)-based algorithm was integrated with Recurrent Neural Networks (RNNs)-based algorithm to diagnose machine faults. The integrated model effectively captured both spatial and temporal features from machine measurements.

Permanent Magnet Synchronous Motors (PMSMs) are extremely popular in industrial applications because of their high-efficiency and large power rating^19^. The machine windings may be damaged if turn-to-turn faults are not promptly detected and segregated^19^. As a consequence, it is indispensable to detect online turn-to-turn faults in order to protect the machine windings. Numerous researches were published to diagnose and analyze the turn-to-turn faults situated on the windings of the electrical AC machine. In^20^, current waves were analyzed using a Fast Fourier Transform (FFT) to figure out the turn-to-turn faults. The number of short-turns and the fault location could be determined by using a low-voltage supply of PMSM excitation at the motor standstill state, along with the resistance and inductance obtained from the current data^21^. In^22^, online fault detectors used the square of the negative-sequence components of the machine measurements. With regard to great security requirements, a Dual-Redundancy Permanent Magnet Synchronous Motor (DRPMSM) can continue to function when a fault takes place on one of the dual windings per phase. Turn-to-turn faults within the DRPMSM were detected using the direct axis voltage measurements and imaginary power^23,24^. However, the method of assessment is complicated under diverse operating loads, and discrete values with a fixed sampling rate lacked the capability of fault diagnosis as the fault indicator oscillates uncommonly at a low machine rotation. In^25^, only inter-turn faults situated within the machine windings were detected by the auto-correlation technique. In^25^, the method was unable to distinguish between the faults inside and outside the machine protection zone and could not classify the different faults. Recently, numerous advanced techniques were employed for diagnosis and analysis of turn-to-turn faults, including but not limited to Improved Vague Support Vector Machine (IVSVM)^26^, Bayesian networks^27^, Support Vector Machines (SVM)^28^, and Parzen window^29^. In^30^, a hybrid CNN-LSTM model was applied to detect machines faults. The fault detection performance was enhanced by using the CNN-based algorithm that used to extract features from the analog measurements and the LSTM-based algorithm that used to capture temporal dependencies. In^31^, an Artificial Neural Network (ANN) was successfully applied to diagnose winding faults. In^32^, an infrared thermography device was used to identify temperatures and hotspots in diverse locations of the windings. Thermography was used in induction motors for monitoring fault condition. The high cost of the infrared apparatus is the primary demerits of the thermal monitoring method.

Stator winding voltages or currents are the most common measurement for machine fault diagnosis, since they are acquired using instrument transformers or probes^33^. The current/voltage-based methods do not require extra sensors, and they are more reliable than the magnetic flux‐based methods^33^. For an AC machine winding with multiple branches, comparing the different branch currents serves as a differential TTF protection^33^. In this approach, the currents in various branches of a stator winding are compared. In a healthy situation, the differential current is nearly zero, while, it increases and circulates between the shorted turns when a winding TTF occurs^33^.

Traditional differential voltage/current protections were based on voltage/current phasors^34^. A critical problem with differential current relays of the longitudinal type arises when turn-to-turn faults happen in the same winding, which leads to a reduced turns due to the short-circuit event^35^. The reduced number of turns involved in the fault decreases the circulating short-circuit currents, which can blind the conventional differential protection. The machine windings protection against earth leakage faults is performed with the restricted earth fault protection, which has a greater sensitiveness than the differential current protection^36,37^. However, the restricted earth fault relay can be used to protect the machine against only grounding and asymmetric faults, which resembles a backup protection for the primary differential protection, and more specified specifications for instrument transformers should be taken into account^36,37^. Another method that is applicable to identify the TTF in machine windings is based on the residual voltage^38^. In order to measure the neutral voltage, an extra open delta voltage transformer is created. In the healthy machine, the sum of the three phase voltages of the open delta voltage transformer is about zero. Whereas, the residual voltage is no longer zero in the case of TTF. In this approach, the sum of the third harmonics is non‐zero, which must be eliminated to avoid undesirable machine tripping^38^. The additional VT is not financially feasible and may cause some installation problems in some cases. To obviate the utilization of additional VT, a zero‐sequence voltage (ZSV) can be calculated in lieu of measuring it^38^. In^38^, a protection scheme was used to compute the zero‐sequence voltage (ZSV) instead of measuring it. Because of the inherent asymmetry of the AC machine, the ZSV is not always zero even in the healthy setting. Thus, a threshold should be taken into account. When the ZSV is greater than the threshold value, a turn-to-turn fault was observed on the winding.

In^39,40^, the two methods aim to detect the faults and measure the asymmetry of the electrical signals taken at the machine load terminals. In^39^, the method was dependent on the alienation estimator for voltage and current waves. In^40^, the coherence coefficients were calculated for the machine waveforms. Therefore, the alienation-based algorithm^39^ takes longer to detect faults than the coherence-based algorithm^40^. In^39,40^, the approaches failed to (1) detect inter-turn faults, (2) distinguish between faults inside and outside the machine protection area, (3) classify the ten shunt faults that are located within the machine protection region, and (4) select the faulty phase(s) of the three-phase machine windings. To address these issues, a smart differential voltage protection based on the coherence coefficients estimated for only voltage signals is proposed. Recently, the coherence algorithm was previously used to establish an automatic power factor corrector (APFC)^41^, a synchronization system^42^, a busbar protection scheme^43^, and a detection and measurement tool for imbalance and disturbance of three-phase generator voltages and currents^44^. Hence, cross-coherence and auto-coherence coefficients for the voltage waves can be used to diagnose faults on the stator windings of the three-phase machines. The proposal requires the three-phase voltage measurements at the midpoint and full terminal of each phase stator winding of the machine. The methodology is empirically validated on a three-phase AC machine. To accomplish extensive experimental tests on various turns and windings of the AC machine, the windings have been re-winded to get 20 taps for each phase. Furthermore, this configuration allows for different turn-to-turn, external, and internal faults with respect to the machine protection zone to be implemented.

The present manuscript mainly consists of the following: the fundamentals of the differential voltage protection based on the coherence technique are amply illustrated in Section “Coherence-based differential voltage protection”. A practical system for examining the protection algorithm is described in Section “Experimental system”. The practical results will be documented and analyzed in Section “Testing results analysis”. In Section “Protection characteristics”, the algorithm’s characteristics are assessed, its merits are discussed, and it is compared to other recent methods. The algorithm’s assumptions are introduced in Section “Algorithm assumptions”, and the main contributions are listed in Section “Contributions”. Finally, the main conclusions are drawn in Section “Conclusions”.

### Proposal characteristics

The test results affirm that a coherence-based differential voltage protection has the following merits:

1. A novel coherence-based technique of differential voltage relay for AC machine windings is presented,
2. The windings of a variety of equipment, such as power transformers and AC machines, can be protected by a modified wiring circuit for the differential voltage protection,
3. The coherence indicators can be used to pinpoint the moment of fault presence,
4. Continuous monitoring of coherence trajectories can be accomplished,
5. The methodology can find turn-to-turn and shunt faults,
6. The algorithm can be integrated with other digital systems to enhance protection redundancy and reliability for protecting the windings of large-scale equipment,
7. A new configuration for tripping curves dependent on both cross-coherence and auto-coherence indicators is developed for the protection method to distinguish between turn-to-turn, external, and internal faults,
8. The technique can be used to classify shunt faults inside the protection zone of the machine using cross-coherence indicators,
9. The algorithm can quantify the tripping time in the incidence of turn-to-turn faults,
10. The ratios of asymmetry and disturbance for the three-phase voltages can be evaluated by utilizing the cross-coherence and auto-coherence indicators, respectively,
11. The advanced approach achieves high rates of speed, security, dependability, reliability, and accuracy,
12. It is possible to protect single-phase or three-phase machine windings with the coherence algorithm,
13. The algorithm sends a tripping permission when turn-to-turn or internal shunt faults occur, while it is restricted in the cases of external faults or normal operations,
14. The protection requirements rely on numerical values of coherence settings and the size of the data window. The coherence settings can be used to set tripping and blocking zones within the proposed characteristic curves of the relay, and the data window is useful for controlling the computation time of the coherence algorithm. Consequently, the approach can modify protection attributes,
15. Arithmetic calculations are not used to determine coherence settings, and.
16. The parameters of electrical machines and power system components are not required for the algorithm to work.

### Generalized comparison

Table 1 compares the developed method with some existing methods used to diagnose different faults situated on the stator windings of the AC machines.

**Table 1:** Comparison between the developed method and multiple existing methods.

Item	Proposed method	Existing methods
1- Required measurements	• Two voltage signals are converted into discrete values to estimate the coherence coefficients, which are measured using two voltage transformers installed at two different points of the same AC machine stator winding. Each stator winding has a center point and a full point for the voltage measurements. As a consequence, data processing and transmission are executed swiftly	• To protect AC machines, most existing protection methods required the measurement of three-phase currents^12,19,34–37^, others needed three-phase voltages and currents^39,40^, and fewer methods necessitated three-phase voltages^33,45^. But certain methods do not require any voltage or current signature^10,11,32^. For three-phase machines, the majority of measurements are acquired at neutral, load/supply, or both terminals
2- Main concept	• The approach relies on the cross-coherence and auto-coherence coefficients, which can be quantified for the two voltage signals for each phase	• Most methods detected the turn-to-turn and shunt faults by estimating the RMS values of current^33–37^, voltage^33^, power^24^, running frequency^20^, impedance, or a combination of these variables^46^, which affect the main parameters of a machine’s power quality
3- Protection tripping characteristic curves	• The external and internal faults can be distinguished using the restraining and tripping regions, respectively, included in the designed quadratic characteristic curves based on the cross-coherence and auto-coherence coefficients, where their values lie between zero and one per unit	• Most relay characteristic curves have an open form, their boundaries are not bounded, and their settings are contingent on the parameters of the AC machines ^33–38^. Moreover, instrument transformer specifications have an impact on the relay setting/characteristics^33–38^
4- Protection settings	• The numerical values of the data window and the coherence settings can be used to modify the attributes of the relay (such as sensitivity, dependability, security, and speed) • No calculation of relay settings is required. Tripping and restraining regions within the characteristic curves can be altered using the coherence settings, and varying a machine size does not affect these settings	• Multiple existing protection techniques need in-depth studies to equip their settings accurately^33–38^ • A variation in the machine size leads to a necessary modification in the relay settings^33–38^
5- Multiple functions	• The coherence coefficients for voltage signals can be used to implement several functions as follows: (1) The identification of shunt faults, (2) The determination of inter-turn faults, and the estimation of their convenient operating times when they are present, (3) The classification of ten shunt fault types, (4) The selection of the phases that are faulty, (5) The discrimination between shunt faults located within and out of the equipment protection zone, and (6) The detection and assessment of the three-phase voltages that are unbalanced,	• Many methods only run one or two protection functions. In^10,11,32,33,36,39,40^, the approaches are unable to perform the following functions: (1) The categorization of ten shunt fault types, (2) he discrimination of the phases that are faulty, and (3) The distinction between shunt faults that happen inside or outside the machine protection zone
6- Response to inter-turn faults	• The proposed scheme responds to the occurrence of inter-turn faults	• Several strategies become inactive when inter-turn faults occur^34–36^
7- Harmonics filtering	• The utilization of the data window concept serves as a digital low-pass filter, removing some ripples and harmonics from voltage measurements	• Some approaches need additional low pass filters to remove undesirable harmonics from input signals to the relay, and relieve the effect of very fast transients in the measurements^33^
8- Protection Redundancy	• The computational technique based on the coherence function reinforces the redundancy and reliability properties of the protection system. This is because it employs both auto-coherence and cross-coherence algorithms	• Several existing techniques lack the protection redundancy and reliability features^10,11,32,36^
9- Protection integration	• The numerical method can be incorporated with other existing protection techniques to boost the protection reliability	• The protection integration characteristic is not involved in multiple techniques, resulting in a lack of the protection reliability^10,11,32^
10- Protection speed	• The developed algorithm is highly responsive	• Some strategies have a remarkable delay in identifying turn-to-turn and shunt faults^39,40^
11- Protection accuracy	• The advanced method exhibits precision in fault detection. The quantitative results illustrate that it can detect most fault situations accurately. Furthermore, it is capable of estimating the suitable tripping time in the case of turn-to-turn faults, depending on its severity	• Several existing methods have a shortage of the accuracy for inter-turn faults detection^10,11,32^
12- Protection security	• The protection security can be enhanced by the excess of the data window or the coherence settings. This extends the blocking area and reduces the tripping area within the developed characteristic curves based on the coherence coefficients, simultaneously	• The security of differential or restricted earth fault current relays can be raised by maximizing the threshold values of the differential current, as well as the restraining factor^10,11^. This will decrease the size of the tripping area and increase the size of the restraining area within their characteristic curves
13- Protection dependability	• The protection dependability can be improved by the reduction in the data window or the coherence settings. This lessens the blocking area and expands the tripping area within the developed characteristic curves at the same time	• The dependability of differential or restricted earth fault current relays can be reinforced by reducing the threshold values of the differential current, as well as the restraining factor^10,11,36^. This will extend the size of the tripping area and reduce the size of the restraining area within their characteristic curves
14- Protection sensitivity	• The reduction of the data window or the coherence setting deviations of the proposed characteristic curves can enhance the protection sensitivity. The setting deviations can be used to alter the blocking and tripping areas situated within the developed characteristic curves, thereby modifying the sensitivity of the protection • Each characteristic curve based on the coherence coefficients has a stationary size and extends from 0.0 to + 1.0 per unit. Thus, if the blocking area is decreased, the tripping area will be increased within each characteristic curve	• The sensitivity of differential or restricted earth fault current relays can be improved by minimizing the threshold values of the differential current, as well as the restraining factor^34–37^. This will increase the tripping area size and decrease the blocking area size within their characteristic curves • Only a single curve can be selected for these relays. Accordingly, the protection sensitivity of these relays cannot be automatically adjusted^34–37^ • The settings calculation of the operating characteristic curves for these relays relies on the parameters of AC machines and the specifications of current transducers^34–37^
15- Various Applications	• The protection algorithm based on the coherence estimators can be applied for a diversity of AC machine types and sizes. It can be used to find diverse types of faults that occur on the windings of the machines with different power and voltage scales. Furthermore, it can be used to create a protection strategy to protect power transformer windings. The method can be effective for protecting single-phase and three-phase machines as well. It can also be applied to protect the AC machines with one or two windings per phase	• Several existing techniques can be used to protect different components of traditional and smart power systems with a wide range of power and voltage ratings. They can be employed to protect AC generators^5,9,31,33,38–40,44^, motors^1–4,6–8,10–15,18–25,30,32,45^ and power transformers^26–29,34–37^ from shunt or inter-turn faults
16- Financial requirements	• The proposal has lower financial commitments than some published methods	• Certain existing approaches necessitate more financial requirements^10,11,32^

## Coherence-based differential voltage protection

The main parameters of a power quality are the frequency, RMS value, phase difference, symmetry, and wave appearance. A fault in the electrical machine windings can cause a change in at least one of these parameters. The coherence technique is an appropriate tool to define a fault emergence and measure the correlation between any two data sets of one or two variable(s)^39,40^. The coherence algorithm can perform the roles of fault detection and unbalance assessment between two data sets using the same arithmetic operation at the same time. In consequence, it can be harnessed to create an effective and reliable scheme for windings protection for multiple electrical machines, such as synchronous generators and motors, induction generators and motors, and power transformers^40,41^. The auto-coherence indicator and cross-coherence indicator are two distinct types of coherence indicators that can be used in this work^42–44^. The proposed method aims to obtain three-phase voltages, which are measured at the midpoints and full ends for the windings of the AC machine. For each phase winding, the VTR_1_ of the voltage transformer (*VTX*_*1*_) at the full end is double the VTR_2_ of the voltage transformer (*VTX*_*2*_) at the midpoint. The auto-coherence and cross-coherence indicators computed for the voltages can be used to detect and discriminate faults in the proposal of differential voltage protection. The cross-coherence indicator represents the main protection algorithm, while the auto-coherence indicator is considered the backup protection algorithm. The cross-coherence indicator, which is estimated between each two voltage signals of the same phase winding of the machine, can be used to identify and classify internal shunt faults in the differential voltage protection. Hence, the cross-coherence indicator resembles a conventional differential voltage relay, where it can compare the similarity between the two voltages, and it can function for every distinct phase. Whereas, the auto-coherence indicator can be used to determine faults in general, including winding-to-winding, winding-to-neutral, and turn-to-turn faults. The subsequent section will delineate the mathematical equations applicable to both types of coherence.

### Coherence indicators

#### Cross-coherence indicator

The cross-coherence indicator can be exploited to quantify the correlation, as a function of frequency, between two different variables. Its amount extends from 0.0 to + 1.0, which is similar to the value per unit^41,42^. The cross-coherence indicator is computed (between each two conforming data sets) for the two voltages (*v*_*1x*_*(n)* and* v*_*2x*_*(n)*) measured for the ‘*X*’ phase of the induction machine stator windings^41,42^. The arithmetic operation is done continuously. The quantity of measurements per the data set corresponds to the quantity of measurements per a single cycle (*N*_*s*_ = *N*_*c*_) of the fundamental power frequency for the machine voltage signals*.* For the three-phase induction motor stator windings, three cross-coherence indicators, namely *Cv*_*12a*_*, Cv*_*12b*_*,* and* Cv*_*12c*_, can be obtained^43,44^. The mathematical formula for the cross-coherence indicator (*Cv*_*12x*_(*k*)) computed between the two voltages (*v*_*1x*_*(n)* and* v*_*2x*_*(n)*) taken at the midpoint and complete terminal of the phase stator winding ‘*X*’ can be expressed as follows^40^:

1 $$Cv_{12x} (k) = \frac{{\left[ {\left( {\sum\limits_{n = 0}^{N - 1} {V_{1x1} (k) \times V_{2x1} (k)} + V_{1x2} (k) \times V_{2x2} (k)} \right)^{2} + \left( {\sum\limits_{n = 0}^{N - 1} {V_{1x1} } (k) \times V_{2x2} (k) - V_{1x2} (k) \times V_{2x1} (k)} \right)^{2} } \right]}}{{\sum\limits_{n = 0}^{N - 1} {\left[ {(V_{1x1} (k))^{2} + (V_{1x2} (k))^{2} } \right]} \times \sum\limits_{n = 0}^{N - 1} {\left[ {(V_{2x1} (k))^{2} + (V_{2x2} (k))^{2} } \right]} }}$$

where $V_{1x1} (k) = \sum\limits_{n = 0}^{N - 1} {\left[ {v_{1x} (n) \cdot \cos \left( {\frac{2\pi kn}{N}} \right)} \right]}$, $V_{1x2} (k) = \sum\limits_{n = 0}^{N - 1} {\left[ {v_{1x} (n) \cdot \sin \left( {\frac{2\pi kn}{N}} \right)} \right]}$, $V_{2x1} (k) = \sum\limits_{n = 0}^{N - 1} {\left[ {v_{2x} (n) \cdot \cos \left( {\frac{2\pi kn}{N}} \right)} \right]}$, $V_{2x2} (k) = \sum\limits_{n = 0}^{N - 1} {v_{2x} (n) \cdot \sin \left( {\frac{2\pi kn}{N}} \right)}$where *V*_*1x1*_*(k)*: The cosine term of the DFT for the voltage signal *v*_*1x*_*(n)*, *V*_*1x2*_*(k)*: The sine term of the DFT for the voltage signal *v*_*1x*_*(n)*, *V*_*2x1*_*(k)*: The cosine term of the DFT for the voltage signal *v*_*2x*_*(n),*and *V*_*2x2*_*(k)*: The sine term of the DFT for the voltage signal *v*_*2x*_*(n),*

#### Auto-coherence indicator

The auto-coherence indicator can be used to figure out the interrelationship, as a function of frequency, between each couple of successive data groups of the same variable. The two data sets are separated by one cycle. Its value can be between 0.0 and + 1.0^39,40^. The auto-coherence indicator can be computed for each voltage wave measured at the midpoint and complete terminal of each stator winding. This estimation process is performed between each two successive data groups that differ in time by a cycle of the nominal power frequency^42,43^. In this study, the presetting data window can be set to a sub-cycle, but it will be taken as a single cycle. This is related to the protection requirements and the prevailing conditions in a power grid. Six voltage signals are acquired from the three-phase windings of the induction machine, leading to six auto-coherence indicators (*Cv*_*1a*_*, Cv*_*1b*_*, Cv*_*1c*_*, Cv*_*2a*_*, Cv*_*2b*_*,* and* Cv*_*2c*_) can be estimated^39,40^. The mathematical equation for the auto-coherence indicator (*Cv*_*1x*_(*k*)) can be formulated for the full voltage (*v*_*1x*_*(n)*) of the phase ‘*X*’ stator winding of the induction motor as follows^40^:

2 $$Cv_{1x} (k) = \frac{{\left[ {\left( {\sum\limits_{n = 0}^{N - 1} {V_{1x1} (k) \times V_{3x1} (k)} + V_{1x2} (k) \times V_{3x2} (k)} \right)^{2} + \left( {\sum\limits_{n = 0}^{N - 1} {V_{1x1} } (k) \times V_{3x2} (k) - V_{1x2} (k) \times V_{3x1} (k)} \right)^{2} } \right]}}{{\sum\limits_{n = 0}^{N - 1} {\left[ {(V_{1x1} (k))^{2} + (V_{1x2} (k))^{2} } \right]} \times \sum\limits_{n = 0}^{N - 1} {\left[ {(V_{3x1} (k))^{2} + (V_{3x2} (k))^{2} } \right]} }}$$

$$V_{3x1} (k) = \sum\limits_{n = 0}^{N - 1} {\left[ {v_{1x} (n - N_{c} ) \cdot \cos \left( {\frac{2\pi kn}{N}} \right)} \right]}$$

$$V_{3x2} (k) = \sum\limits_{n = 0}^{N - 1} {\left[ {v_{1x} (n - N_{c} ) \cdot \sin \left( {\frac{2\pi kn}{N}} \right)} \right]}$$

where *V*_*3x1*_*(k)*: The cosine term of the DFT for the voltage wave *v*_*1x*_*(n-N*_*c*_*)*, and *V*_*3x2*_*(k)*: The sine term of the DFT for the voltage wave *v*_*1x*_*(n-N*_*c*_*)*.

Moreover, the mathematical equation for the auto-coherence (*Cv*_*2x*_(*k*)) can be computed for the midpoint voltage (*v*_*2x*_*(n)*) of the phase ‘*X*’ stator winding of the induction motor, as given below^40^.

3 $$Cv_{2x} (k) = \frac{{\left[ {\left( {\sum\limits_{n = 0}^{N - 1} {V_{1x1} (k) \times V_{4x1} (k)} + V_{1x2} (k) \times V_{4x2} (k)} \right)^{2} + \left( {\sum\limits_{n = 0}^{N - 1} {V_{1x1} } (k) \times V_{4x2} (k) - V_{1x2} (k) \times V_{4x1} (k)} \right)^{2} } \right]}}{{\sum\limits_{n = 0}^{N - 1} {\left[ {(V_{1x1} (k))^{2} + (V_{1x2} (k))^{2} } \right]} \times \sum\limits_{n = 0}^{N - 1} {\left[ {(V_{4x1} (k))^{2} + (V_{4x2} (k))^{2} } \right]} }}$$

$$V_{4x1} (k) = \sum\limits_{n = 0}^{N - 1} {\left[ {v_{2x} (n - N_{c} ).\cos \left( {\frac{2\pi kn}{N}} \right)} \right]}$$

$$V_{4x2} (k) = \sum\limits_{n = 0}^{N - 1} {\left[ {v_{2x} (n - N_{c} ) \cdot \sin \left( {\frac{2\pi kn}{N}} \right)} \right]}$$

where *V*_*4x1*_*(k)*: The cosine term of the DFT for the voltage wave *v*_*2x*_*(n-N*_*c*_*)*, and *V*_*4x2*_*(k)*: The sine term of the DFT for the voltage wave *v*_*2x*_*(n-N*_*c*_*)*.

The subscript* X* is the designated phase *A, B,* or *C*.

### Faults detection and localization

Table 2 includes the operation conditions of the coherence technique and its behavior. The machine condition is what determines the protection action.

**Table 2:** The operation conditions of the coherence technique and its behavior.

Sr. No	Coherence indicators range for phase *X* winding	Phase ‘*X’* winding state
	The presetting divergences (*Δx*_*1*_ and* Δx*_*2*_) of the coherence are 0.05	
1	*1.0*—*Δx*_*1*_ ≤ *Cv*_*12x*_ ≤ *1.0,* and *1.0*—*Δx*_*2*_ ≤ *Cv*_*1x*_ ≤ *1.0,* and *1.0*—*Δx*_*2*_ ≤ *Cv*_*2x*_ ≤ *1.0*	Normal operation or external fault for phase *X*
		Blocking order for the machine breakers
2	*Cv*_*12x*_ < *1.0—Δx*_*1*_	Internal shunt fault for phase *X*
		Tripping permission for the machine breakers
3	*1.0*—*Δx*_*1*_ ≤ *Cv*_*12x*_ ≤ *1.0,* and *0.0* ≤ *Cv*_*1x*_ < *1.0*—*Δx*_*2*_*,* or *0.0* ≤ *Cv*_*2x*_ < *1.0*—*Δx*_*2*_	External fault, or Turn-to-turn fault for phase *X*
	Blocking order for the machine breakers in the state of external faults for phase *X*	Tripping permission for the machine breakers in the incidence of turn-to-turn faults for phase *X*

### Tripping curve based on coherence

**Fig. 1:**
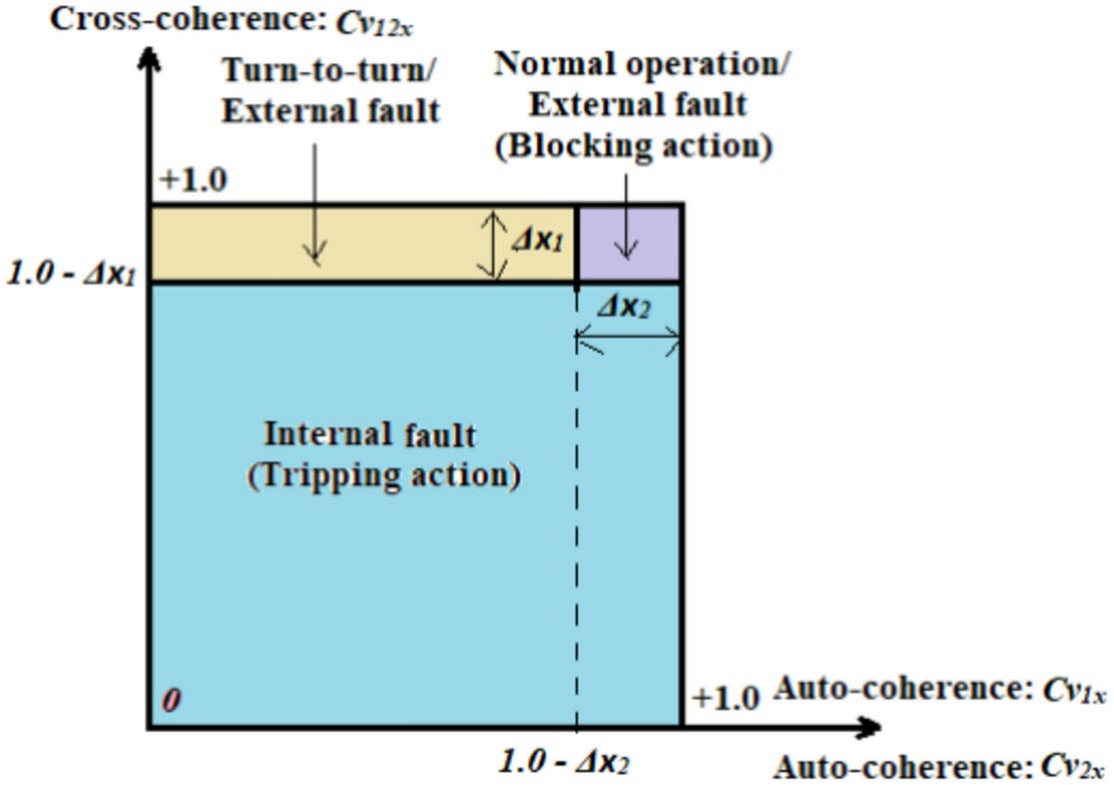
Tripping curve based on single-phase coherence indicators (*Cv*_*12x*_, *Cv*_*1x*_, and *Cv*_*2x*_).

The computational technique works separately for each phase of the IM stator winding. The tripping curve, which is derived from the single-phase coherence factors (*Cv*_*12x*_*, Cv*_*1x*_*,* and *Cv*_*2x*_), is depicted in Fig. 1. It has a square form, and it can be partitioned into three areas that are defined below:

1. The normal operation/external fault area: The blocking order is issued to prevent isolating the induction machine circuit breakers,
2. The turn-to-turn fault/external fault area: The tripping permission is sent to separate the induction machine circuit breakers when the turn-to-turn faults occur; whereas, the blocking order is issued in the state of external faults, and
3. The internal shunt fault area: The tripping permission is sent to disconnect the induction machine circuit breakers to protect it from the internal faults.

Table 3 includes the operating time of the algorithm for different machine conditions. The table demonstrates that the operating time is infinite in normal operating conditions and instantaneous when internal faults occur. To ensure the turn-to-turn fault occurrence, the actual tripping time of the protection algorithm should initiate after the starting time of the motor.

**Table 3:** The operating time of the coherence algorithm.

Machine state	Coherence indicators range for phase ‘*X’* winding	Algorithm operating time (s)
1- Normal operations/ External faults	*1.0—Δx*_*1*_ ≤ *Cv*_*12x*_ ≤ *1.0,* and *1.0 – Δx*_*2*_ ≤ *Cv*_*1x*_ ≤ *1.0,* and *1.0 – Δx*_*2*_ ≤ *Cv*_*2x*_ ≤ *1.0,* and *Kv*_*x*_ ≥ *Kv*_*pu*_	Infinity
2- Internal shunt faults	*Cv*_*12x*_ < *1.0—Δx*_*1*_*, or* *Kv*_*x*_ < *Kv*_*pu*_	Instantaneous
3- Turn-to-turn faults	*1.0—Δx*_*1*_ ≤ *Cv*_*12x*_ ≤ *1.0,* and *0.0* ≤ *Cv*_*1x*_ < *1.0 – Δx*_*2*_*,* or 0.0 ≤ Cv_2x_ < 1.0 – Δx_2_*,* or *Kv*_*x*_ < *Kv*_*pu*_	*Tv*_*op*_

To estimate the tripping time (*Tv*_*op*_) in the incidence of turn-to-turn fault, the following expression can be applied. The formula requires the coefficient *(Kv*_*x*_*)* computed using the peaks’ ratio of the two voltage signals (*v*_*1x*_ and *v*_*2x*_) for phase *X*, the pickup ratio (*Kv*_*pu*_), and the time multiplier (*K*_*s*_).

4 $$Tv_{op} = \frac{Ks}{{\left( {\left( {\frac{{Kv_{pu} }}{{Kv_{x} }}} \right) - 1} \right)}}$$

5 $$Kv_{x} = \left| {\frac{{\min \left\{ {v_{1xpeak} ,v_{2xpeak} } \right\}}}{{\max \left\{ {v_{1xpeak} ,v_{2xpeak} } \right\}}}} \right|$$

where *Tv*_*op*_: The quantified tripping time of the algorithm (s), *K*_*s*_: The time multiplier (in this algorithm, *K*_*s*_ is selected 0.1), *Kv*_*pu*_: The predetermined pickup ratio of the relay (in this algorithm, *Kv*_*pu*_ is selected 0.95), *Kv*_*x*_: The coefficient computed using the peaks’ ratio of the two voltage signals (*v*_*1x*_ and *v*_*2x*_) for phase* X*, *v*_*1xpeak*_: The measured peak value of the voltage signal *v*_*1x*_* (n),* and *v*_*2xpeak*_: The measured peak value of the voltage signal *v*_*2x*_* (n).*

### Protection strategy

**Fig. 2:**
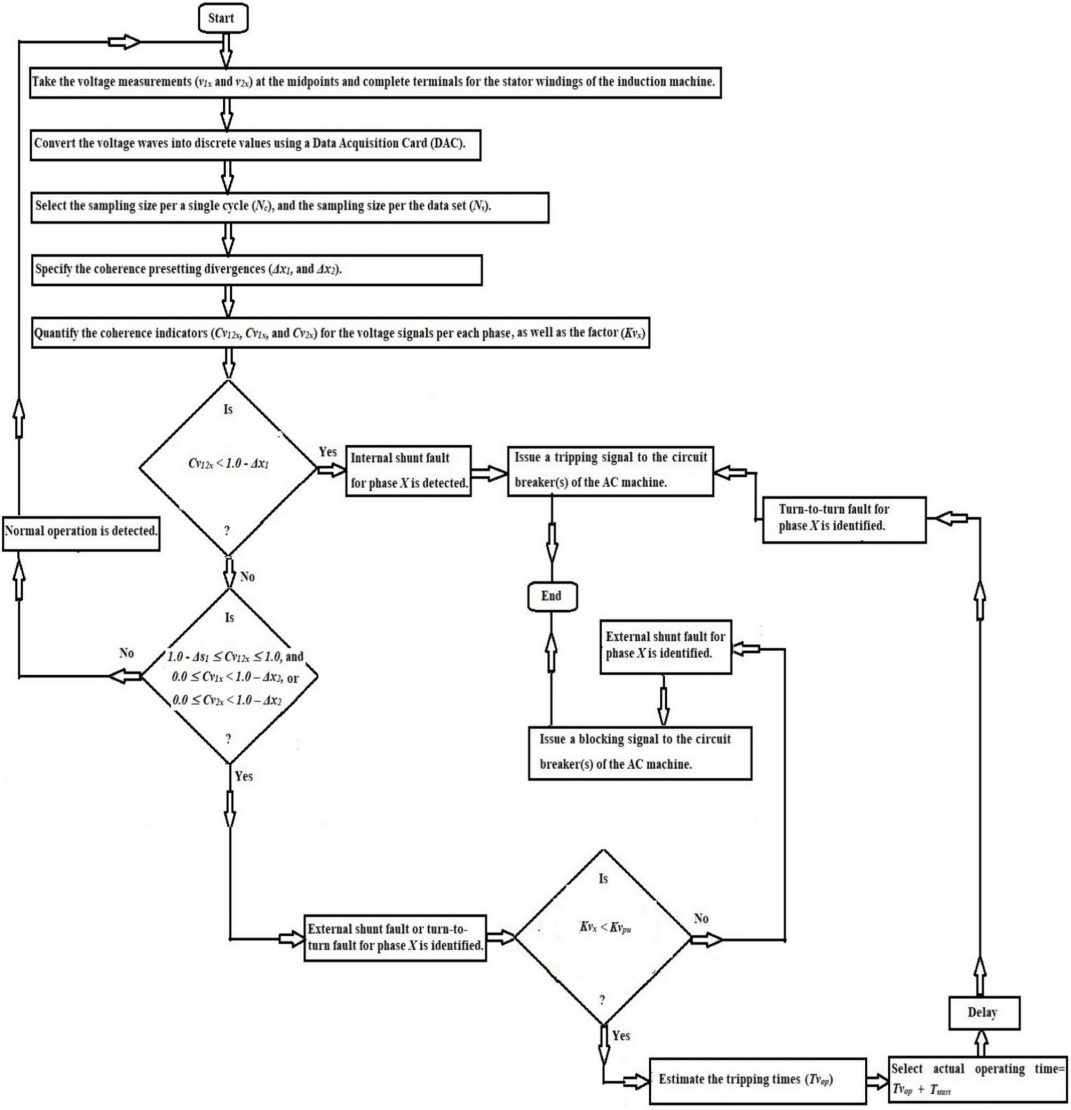
Flow chart of the coherence-based fault detection.

The method mechanism for identifying and distinguishing turn-to-turn, external and internal faults is illustrated in Fig. 2. The protection procedure can be executed for each phase as given below.

**Fig. 3:**
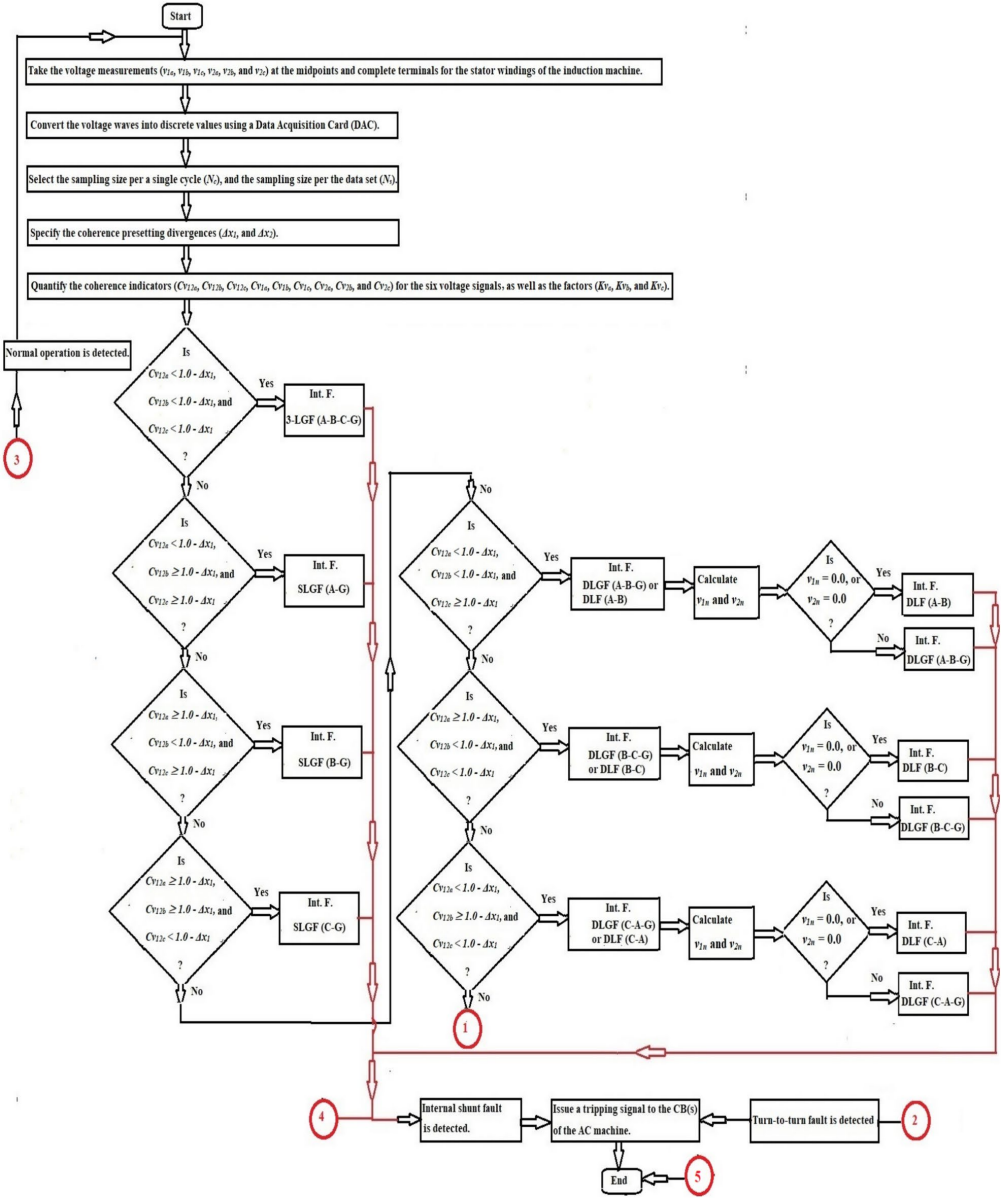

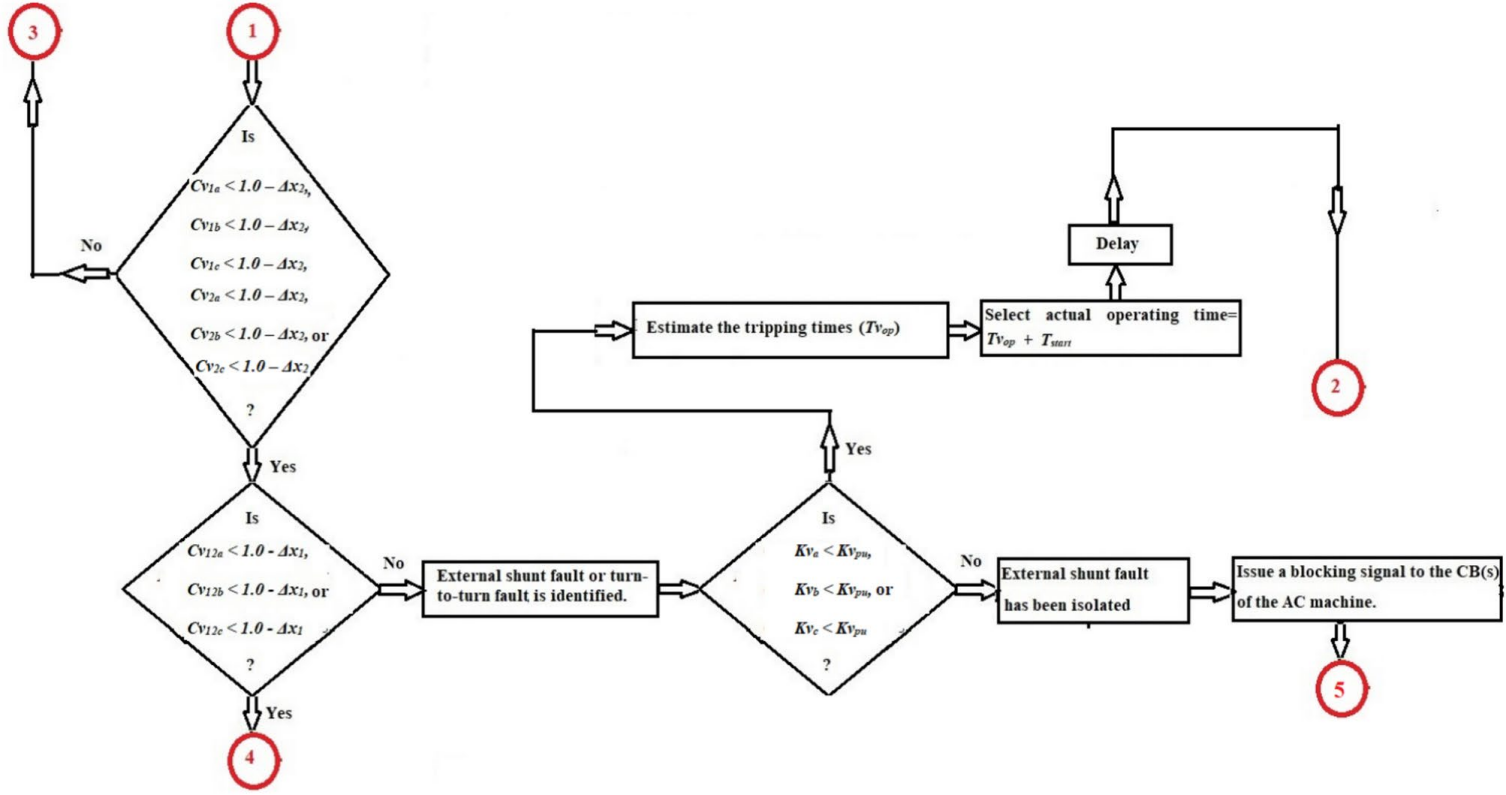
(**a**) Flow chart of the coherence-based fault classification. (**b**) Flow chart of the coherence-based fault classification (Continued).

1. Take the voltage measurements (*v*_*1x*_ and *v*_*2x*_) at the midpoints and supply terminals for the stator windings of the induction machine,
2. Convert the voltage waves into discrete values using a Data Acquisition Card (DAC),
3. Select the sampling size for a single cycle (*N*_*c*_), and the sampling size for the data set (*N*_*s*_),
4. Specify the coherence presetting divergences (*Δx*_*1*_*,* and *Δx*_*2*_). Where,
  *Δx*_*1*_ is the presetting divergence of the cross-coherence indicator (*Cv*_*12x*_), and.
  *Δx*_*2*_ is the presetting divergence of the auto-coherence indicators (*Cv*_*1x*_ and* Cv*_*2x*_),
5. Quantify the phase coherence indicators (*Cv*_*12x*_*, Cv*_*1x*_*,* and *Cv*_*2x*_) for the voltage signals, and the coefficient (*Kv*_*x*_),
6. Execute the operation conditions of the coherence methodology for each phase (as presented in Table 2 and the flow chart in Fig. 2),
7. Compute the tripping time (*Tv*_*op*_) in the incidence of turn-to-turn fault,
8. Classify the shunt faults existing within the machine protection zone (as shown in Fig. 3a,b),
9. Carry out the following protection actions:
  - *Inactive action* Block the machine breakers in the state of healthy phase *X*,
  - *Inactive action* Block the machine breakers in the state of external faults for the phase *X*,
  - *Active action* Trip the machine breakers in the state of internal shunt faults for the phase *X,* and.
  - *Active action* Trip the machine breakers in the state of turn-to-turn faults for the phase *X*.
10. Originate a tripping permission for the AC machine breakers when the machine is prone to internal shunt faults or turn-to-turn faults, and
11. To differentiate between ground and non-ground faults, calculate the neutral voltage (*v*_*1n*_ and *v*_*2n*_) and apply the following condition: If *v*_*1n*_ = *v*_*1a*_ + *v*_*1b*_ + *v*_*1c*_ = *0.0,* or *v*_*2n*_ = *v*_*2a*_ + *v*_*2b*_ + *v*_*2c*_ = *0.0,* the fault is then free from the ground. Otherwise, the fault is through the ground.

### Algorithm speed

The computation time and fault detection speed depend on the size of the selected data set applied to estimate the coherence coefficients. The computation time can be modified depending on the data set. It can be equal to or less than one cycle of the nominal power frequency. Its quantity is affected by the protection requirements and the operating conditions of the machine. Furthermore, the response speed of the digital protection has an immediately interrelated and interdependent relationship with the following items:

1. Its microprocessor speed,
2. The load of computer programming, and
3. The sophistication level of the computer programming.

### Coherence settings criteria

In this work, the coherence presetting values have been investigated on a new setup for an induction motor with tapping three-phase windings. Practically, the coherence threshold values have been modified and verified by seeing the relay response signal and annunciator flag, which provides information on the state of the electrical model to indicate that the protection system is active or non-active. LABVIEW program has been used to adjust the numerical values of the coherence settings, and bypass acceptable imbalances and normal operations. The settings can be easily modified with other experimental models, depending on how much unbalance of the three-phase voltages is acceptable. This is a considerable benefit that can boost the reliability of the suggested technique.

Moreover, the coherence settings possess the capability to regulate the requirements of the protection properties, comprising sensitivity, security, dependability, and speed. Using a feasible data sets and coherence presetting values can actually mitigate the impacts of instrumentation errors, transient faults, and light harmonics. Consequently, the protection response is conditioned on the actual values of the coherence indicators and the coherence pickup values of the protection system.

### Protection algorithm sensitivity

The sensitivity of the coherence algorithm is closely associated with the actual coherence indicators *(Cv*_*12x*_*, Cv*_*1x*_ and *Cv*_*2x*_*)* and the coherence threshold value (*C*_*vpu*_*)*, as shown in Eqs. (6–8).

6 $$Sensitivity\;\alpha \frac{{C_{vpu} }}{{Cv_{12x} }} = \frac{{(1.0 - \Delta x_{1} )}}{{Cv_{12x} }}$$

7 $$Sensitivity\;\alpha \frac{{C_{vpu} }}{{Cv_{1x} }} = \frac{{(1.0 - \Delta x_{2} )}}{{Cv_{1x} }}$$

8 $$Sensitivity\alpha \frac{{C_{vpu} }}{{Cv_{2x} }} = \frac{{(1.0 - \Delta x_{2} )}}{{Cv_{2x} }}$$

Additionally, the sensitivity of the second algorithm depends on the pickup ratio (*Kv*_*pu*_), and the coefficient *(Kv*_*x*_*)* by comparing the peaks’ ratio of the two voltage signals (*v*_*1x*_ and *v*_*2x*_) for phase *X*, as illustrated in Eq. (9).

9 $$Sensitivity\;\alpha \frac{{K_{vpu} }}{{Kv_{x} }}$$

## Experimental system

**Fig. 4:**
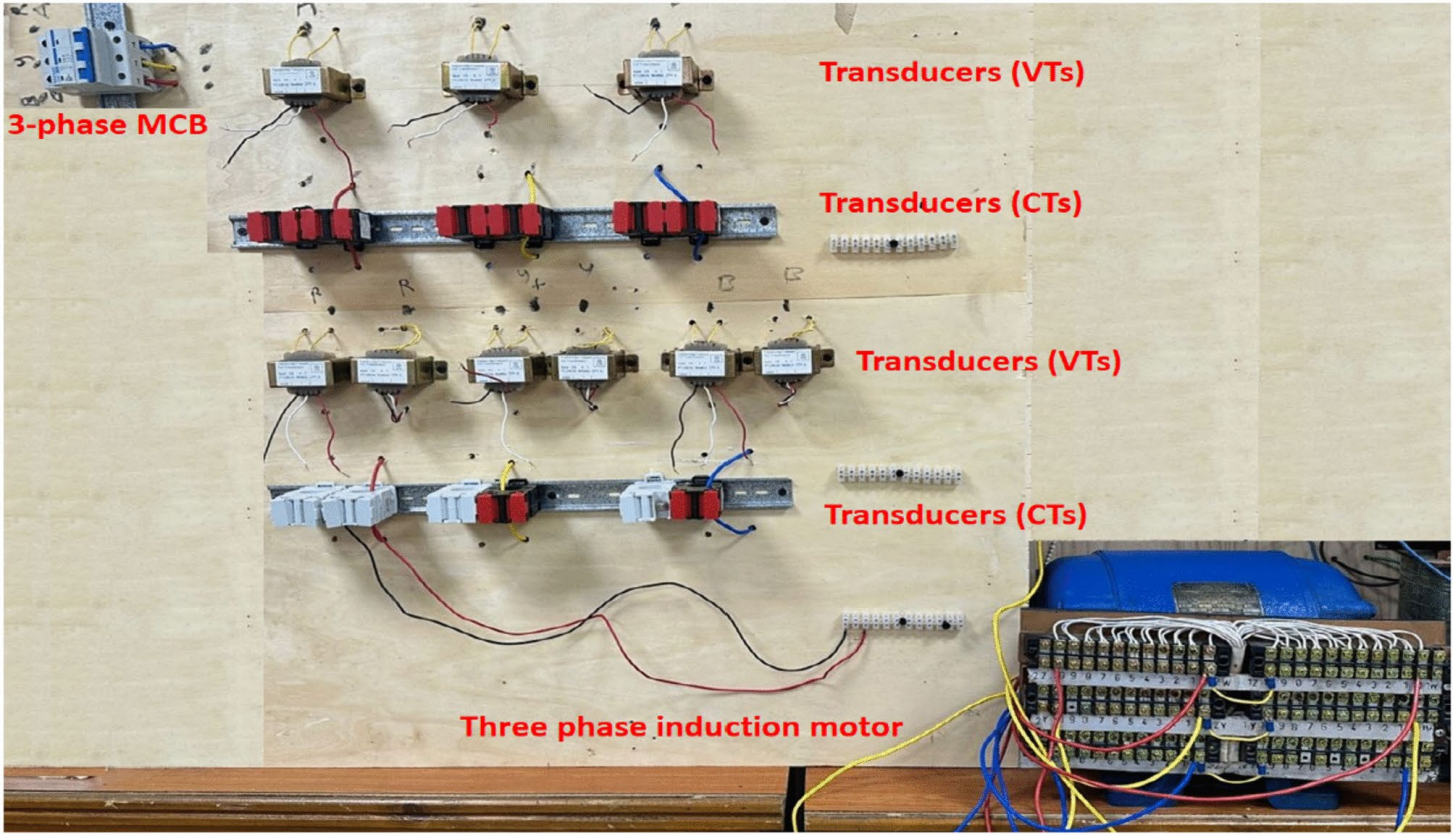

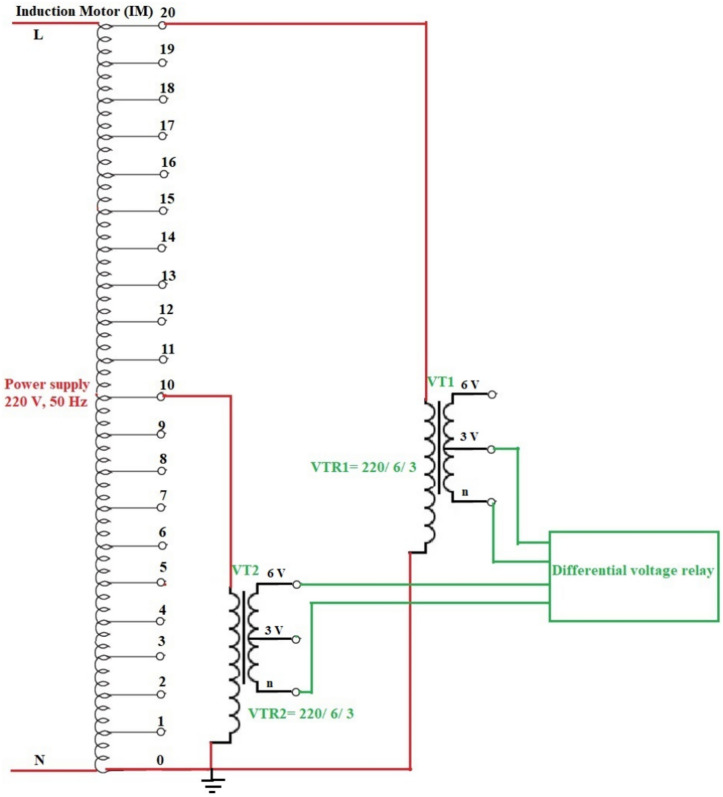
(**a**) Experimental system under test. (**b**) The wiring connection diagram of the experimental system.

The experimental system includes a three-phase power supply with a line voltage rating of 380.0 V, a three-phase supply miniature circuit breaker (CB) with a current rating of 63.0 A, and an induction motor (IM) with a power rating of 2.90 kW. For each stator winding of the AC machine, a voltage transformer (VTX_2_) with a turns’ ratio of 220/6 is installed at the midpoint of the winding, and a second voltage transformer (VTX_1_) with a turns’ ratio of 220/3 is built at the complete terminal of the winding. The three-phase stator windings of the machine have been re-wounded, where 20 taps per each phase winding have been created. This taps configuration aims to construct voltage transformers at the midpoints and complete terminals of the machine stator windings, and to facilitate testing comprehensive experiments to verify the effectiveness of the coherence technique. Diverse scenarios of shunt and series faults on the motor windings will be examined and analyzed, including winding-to-winding, winding-to-neutral, and turn-to-turn faults. A plot of the three-phase induction machine with 20 taps per phase winding is depicted in Fig. 4a. The wiring connection diagram of the induction motor, comprising of 20 taps per phase stator winding, two voltage transducers per each phase, a conversion tool for analog-to-digital signals, and a LABVIEW program accessible on a personal laptop, is shown in Fig. 4b. The parameters’ specifications for the experimental system elements are enumerated in Appendix 1. This project has been undertaken in the electrical power laboratory at Misr University for Science and Technology (MUST) in Egypt.

## Testing results analysis

In this article, the analog-to-digital converter and the LABVIEW package are utilized to test the differential voltage protection under numerous kinds of external, internal and turn-to-turn faults. The USB-6009 analog-to-digital converter, manufactured by National Instruments, is used to convert AC voltage signals into discrete values. The converter will be set to run in differential mode with a sampling frequency of 2.5 kHz. Two voltage transducers are used for each phase to measure the voltage waveforms, which resemble the analog input to the converter, and the LABVIEW platform is used as a monitor screen for the machine status. This implies that six voltage transducers are set up for the three-phase machine stator windings. The voltage waveforms taken from the two voltage transducers are used to obtain the three coherence indicators (*Cv*_*12x*_*, Cv*_*1x*_, and *Cv*_*2x*_) for each phase winding ‘*X*’. Appendix 2 contains input data for the developed technique. In this section, the results for phase winding ‘*A*’ of the motor will be presented. Table 4 contains the output findings for different scenarios of faults. The table also displays the types of protection response.

**Table 4:** Different faults and the types of protection response.

Case study no	Experimental system state	Protection action (Active/Inactive)	Action type (Correct/Incorrect)	Tripping time (s)
Case 1	Normal operation (Phase A)	Inactive	Correct	∞
Case 2	Internal shunt fault (A11–B11)	Active	Correct	Instantaneous
Case 3	Internal shunt fault (A11–C11)	Active	Correct	Instantaneous
Case 4	Internal shunt fault (A12–B12)	Active	Correct	Instantaneous
Case 5	Internal shunt fault (A12–C12)	Active	Correct	Instantaneous
Case 6	Internal shunt fault (A13–B13)	Active	Correct	Instantaneous
Case 7	Internal shunt fault (A13–C13)	Active	Correct	Instantaneous
Case 8	Internal shunt fault (A14–B14)	Active	Correct	Instantaneous
Case 9	Internal shunt fault (A15–C15)	Active	Correct	Instantaneous
Case 10	Turn-to-turn fault (A1–A3)—Series fault	Active	Correct	2.060 s
Case 11	Turn-to-turn fault (A1–A4)—Series fault	Active	Correct	0.666 s
Case 12	Turn-to-turn fault (A1–A5)—Series fault	Active	Correct	0.236 s
Case 13	Turn-to-turn fault (A1–A7)—Series fault	Active	Correct	0.128 s
Case 14	External shunt fault (A5–B5)	Inactive	Correct	∞
Case 15	External shunt fault (A6–B6)	Inactive	Correct	∞
Case 16	External shunt fault (A7–B7)	Inactive	Correct	∞

### Case 1: normal operation (phase A)

**Fig. 5:**
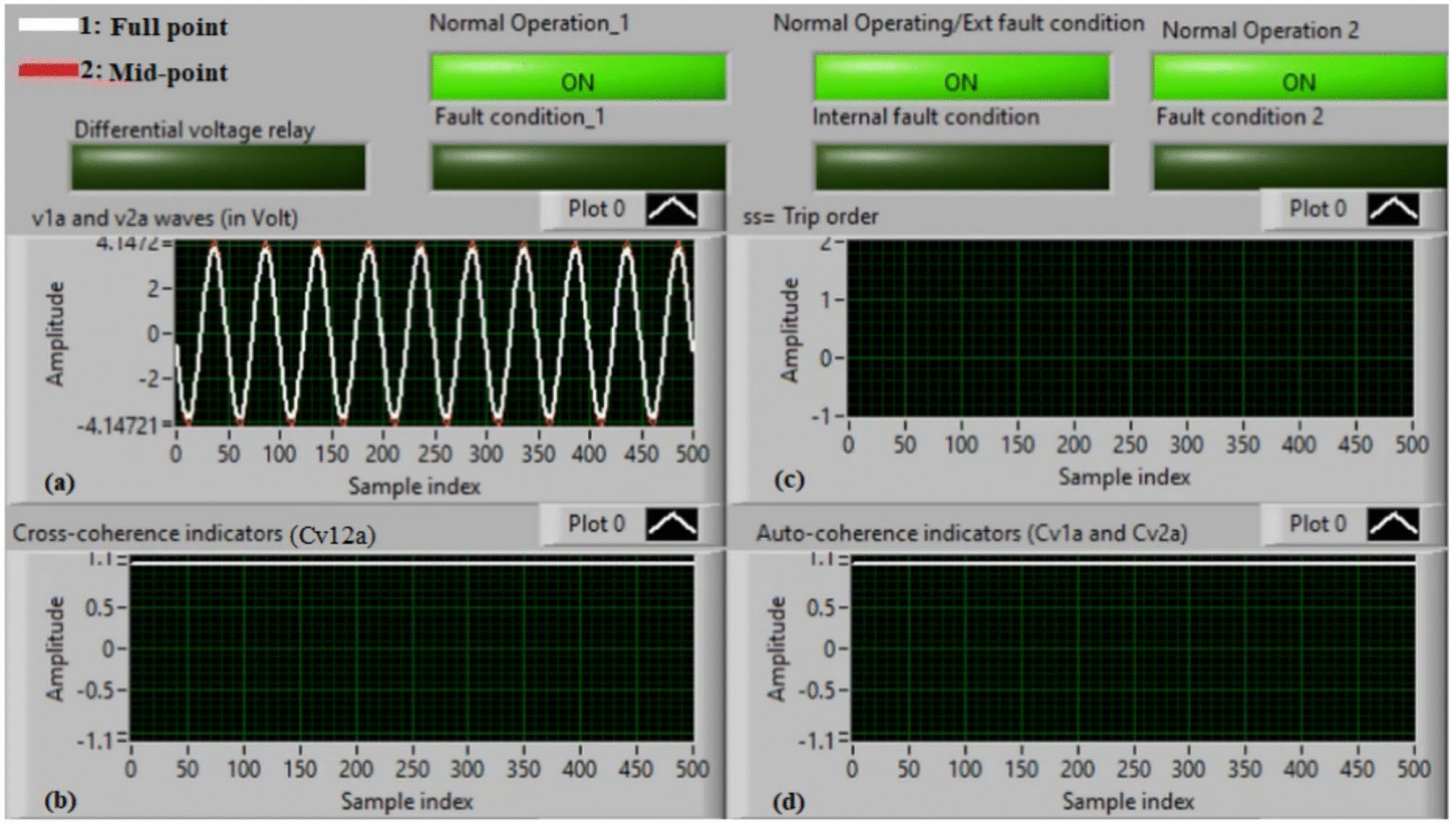
Results for case 1. (**a**) Two measured voltages (v_1a_ and v_2a_), (**b**) Cross-coherence indicator (Cv_12a_), (**c**) Blocking signal, and (**d**) Auto-coherence indicators (Cv_1a_ and Cv_2a_).

Figure 5a–d illustrate the experimental results for case 1. This instance is healthy phase *A*. The two voltages (*v*_*1a*_ and *v*_*2a*_) of the phase *A* stator winding are presented in Fig. 5a. The cross-coherence indicator (*Cv*_*12a*_) calculated between the two phase *A* voltages is depicted in Fig. 5b. Figure 5c shows the blocking signal, which signifies a zero value during the full display time. Figure 5d describes the auto-coherence indicators (*Cv*_*1a*_ and *Cv*_*2a*_) quantified for the two voltages of the phase *A*. As depicted in Fig. 5, the obtained findings indicate that the equipment state is normal. It is observed that the two voltage waves (*v*_*1a*_ and *v*_*2a*_) are identical, and there is no voltage difference between them. The values of the coherence indicators (*Cv*_*12a*_*, Cv*_*1a*_ and *Cv*_*2a*_) are roughly unity. This results in the differential voltage protection based on the coherence indicators being constrained in this test. Consequently, the tripping time of the protection algorithm is infinite.

The experiments on internal shunt faults, spanning from case number 2 to case number 9), will be discussed below.

### Case 2: internal shunt fault (A11–B11)

**Fig. 6:**
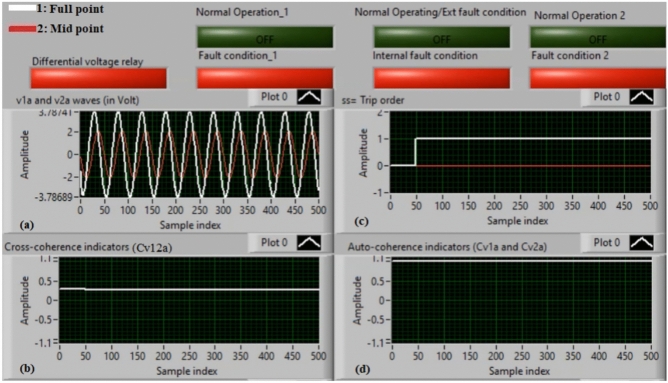
Results for case 2. (**a**) Two measured voltages (v_1a_ and v_2a_), (**b**) Cross-coherence indicator (Cv_12a_), (**c**) Tripping signal, and (**d**) Auto-coherence indicators (Cv_1a_ and Cv_2a_).

Figure 6a–d manifest the experimental results for case 2. Case 2 involves an internal shunt fault (*A11–B11*). Figure 6a exhibits the two voltages (*v*_*1a*_ and *v*_*2a*_) of the phase *A* stator winding for the machine. Figure 6b exhibits the cross-coherence indicator *(Cv*_*12a*_) quantified between the two voltages of phase *A*. Figure 6c depicts the protection tripping signal, and Fig. 6d displays the auto-coherence indicators (*Cv*_*1a*_ and *Cv*_*2a*_) computed for the two voltages of phase *A*. The results of the shunt internal fault (*A11–B11*) for the machine windings are illustrated in Fig. 6. The two voltages (*v*_*1a*_ and *v*_*2a*_) are dissimilar in the presence of the internal shunt fault, where there is a difference between the voltages. In this test, the quantities of the coherence indicators (*Cv*_*12a*_*, Cv*_*1a*_ and *Cv*_*2a*_) are nearly + 0.30, + 0.95 and + 0.95, respectively, during the internal fault occurrence. As a result, the developed approach based on the coherence statistic is active because of the presence of internal fault (which happens between the two phases *A* and* B* windings at tap 11). It is clear that the cross-coherence amplitude (*Cv*_*12a*_) is close to + 0.30, which assures the internal fault event*.* In experiment 2, the protection tripping time is instantaneous.

### Case 3: internal shunt fault (A11–C11)

**Fig. 7:**
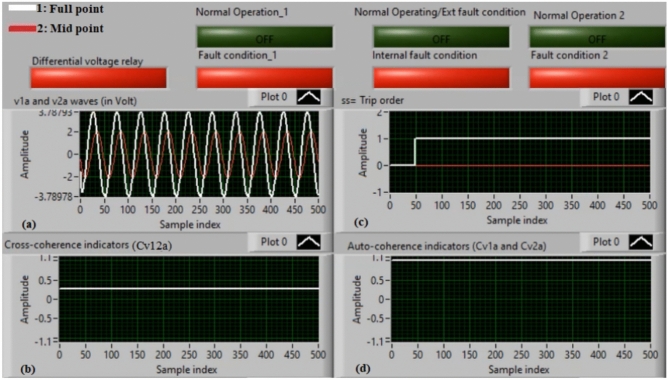
Results for case 3. (**a**) Two measured voltages (v_1a_ and v_2a_), (**b**) Cross-coherence indicator (Cv_12a_), (**c**) Tripping signal, and (**d**) Auto-coherence indicators (Cv_1a_ and Cv_2a_).

Figure 7a–d show the experimental results for case 3. Case 3 is an internal shunt fault (*A11–C11*). Figure 7a exhibits the two voltages (*v*_*1a*_ and *v*_*2a*_) of the phase *A* for the machine, Fig. 7b presents the cross-coherence indicator (*Cv*_*12a*_). Figure 7c depicts the protection tripping signal, and Fig. 7d introduces the auto-coherence indicators (*Cv*_*1a*_ and *Cv*_*2a*_) estimated for the two voltages of phase *A*. The results of the machine stator windings during the internal fault (*A11–C11*) are shown in Fig. 7. The two voltages (*v*_*1a*_ and *v*_*2a*_) differ in the presence of the internal fault, resulting in a differential voltage in the relay. In this case, the amounts of the coherence indicators (*Cv*_*12a*_*, Cv*_*1a*_ and *Cv*_*2a*_) are roughly + 0.30, + 0.95 and + 0.95, respectively, during the fault time. Thus, the present approach is active due to the occurrence of internal fault (which occurs between the two phases *A* and* C* windings at tap 11). It is evident that the cross-coherence amplitude (*Cv*_*12a*_) is close to + 0.30, indicating the internal fault situation. In test 3, the protection tripping time is instantaneous.

### Case 4: internal shunt fault (A12–B12)

**Fig. 8:**
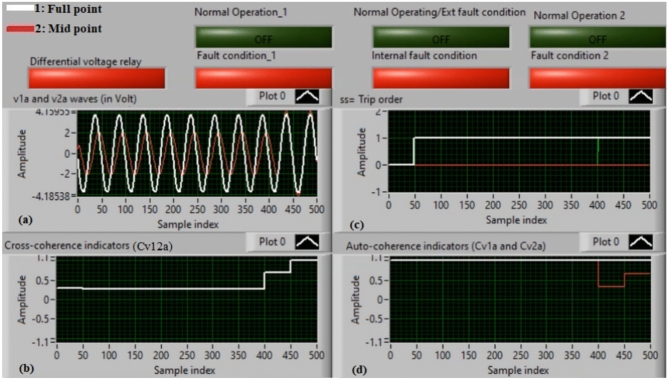
Results for case 4. (**a**) Two measured voltages (v_1a_ and v_2a_), (**b**) Cross-coherence indicator (Cv_12a_), (**c**) Tripping signal, and (**d**) Auto-coherence indicators (Cv_1a_ and Cv_2a_).

Figure 8a–d demonstrate the experimental results for case 4. Case 4 is an internal shunt fault (*A12–B12*). Figure 8a exhibits the two voltages (*v*_*1a*_ and *v*_*2a*_) for the equipment. The cross-coherence indicator (*Cv*_*12a*_) is presented in Fig. 8b. The relay tripping signal is depicted in Fig. 8c, and the auto-coherence indicators (*Cv*_*1a*_ and *Cv*_*2a*_) are introduced in Fig. 8d. The results of the machine stator windings during the internal fault (*A12–B12*) are shown in Fig. 8. The two voltages (*v*_*1a*_ and *v*_*2a*_) are different during the period of internal fault, creating a differential voltage in the protective relay. In this case, the values of the coherence indicators (*Cv*_*12a*_*, Cv*_*1a*_ and *Cv*_*2a*_) are approximately + 0.25, + 0.97 and + 0.30, respectively, during the fault span. This causes the present approach to be active during the extent of internal fault. It is obvious that the cross-coherence amplitude (*Cv*_*12a*_) is close to + 0.25, which confirms the internal fault incidence*.* Consequently, the protection sends a tripping permission, and its operating time is instantaneous.

### Case 5: internal shunt fault (A12–C12)

**Fig. 9:**
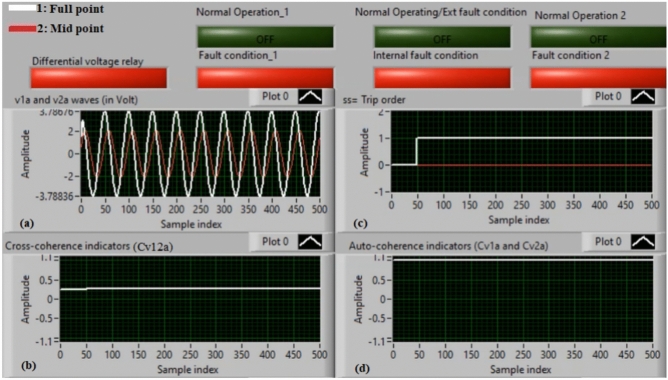
Results for case 5. (**a**) Two measured voltages (v_1a_ and v_2a_), (**b**) Cross-coherence indicator (Cv_12a_), (**c**) Tripping signal, and (**d**) Auto-coherence indicators (Cv_1a_ and Cv_2a_).

Figure 9a–d show the experimental results for case 5. Case 5 is an internal shunt fault (*A12–C12*). Figure 9a exhibits the two voltages (*v*_*1a*_ and *v*_*2a*_) for the phase *A* stator winding of the machine. Figure 9b presents the cross-coherence indicator *(Cv*_*12a*_). Figure 9c depicts the protection tripping signal, and Fig. 9d introduces the auto-coherence indicators (*Cv*_*1a*_ and *Cv*_*2a*_). The results of the phase *A* stator winding during the internal fault (*A12–C12*) are shown in Fig. 9. The two voltages (*v*_*1a*_ and *v*_*2a*_) originate a differential voltage during the internal fault period. In this experiment, the values of the coherence indicators (*Cv*_*12a*_*, Cv*_*1a*_ and *Cv*_*2a*_) are about + 0.22, + 0.97 and + 0.97, respectively, when the internal fault occur. Thus, the present technique is active because of the occurrence of internal fault. It is apparent that the cross-coherence quantity (*Cv*_*12a*_) is close to + 0.22, which affirms the internal fault condition*.* Therefore, the relay operating time is instantaneous in case 5.

### Case 6: internal shunt fault (A13–B13)

**Fig. 10:**
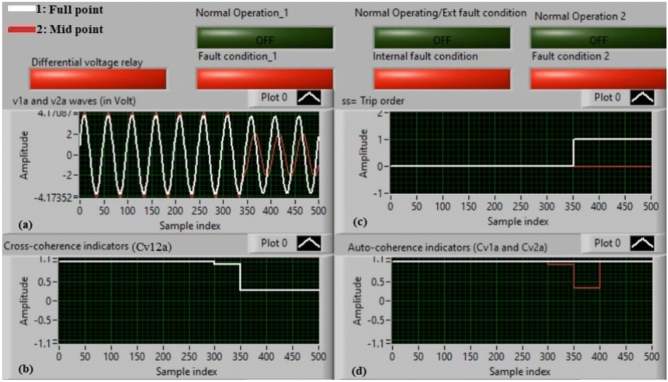
Results for case 6. (**a**) Two measured voltages (v_1a_ and v_2a_), (**b**) Cross-coherence indicator (Cv_12a_), (**c**) Tripping signal, and (**d**) Auto-coherence indicators (Cv_1a_ and Cv_2a_).

Figure 10a–d illustrate the practical results for case 6. Case 6 is an internal shunt fault (*A13–B13*). Figure 10a demonstrates the two voltages (*v*_*1a*_ and *v*_*2a*_). Figure 10b displays the cross-coherence indicator (*Cv*_*12a*_). Figure 10c offers the protection tripping signal, and Fig. 10d presents the auto-coherence indicators (*Cv*_*1a*_ and *Cv*_*2a*_). The results of the phase *A* stator winding of the machine during the internal fault (*A13–B13*) are introduced in Fig. 10. The two voltages (*v*_*1a*_ and *v*_*2a*_) create a differential voltage in the relay due to the internal fault existence. In this incidence, the values of the coherence indicators (*Cv*_*12a*_*, Cv*_*1a*_ and *Cv*_*2a*_) are approximately + 0.20, + 0.97 and + 0.30, respectively, during the internal fault time. Thereby, the occurrence of the internal fault causes the algorithm to become active. It is seen that the value of cross-coherence (*Cv*_*12a*_) is nearly + 0.20, which signifies the internal fault situation*.* As a result, the protection algorithm issues a tripping action to the machine, and its operating time is instantaneous in test 6.

### Case 7: internal shunt fault (A13–C13)

**Fig. 11:**
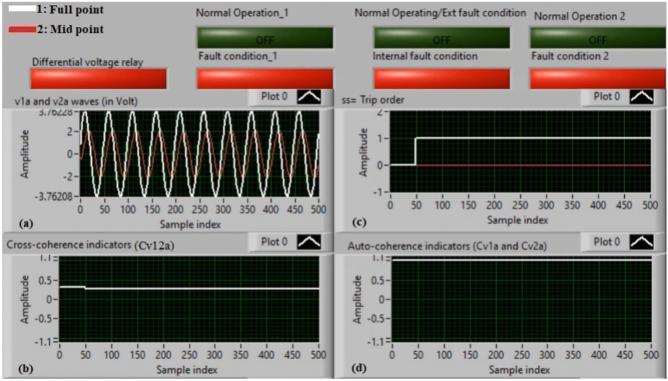
Results for case 7. (**a**) Two measured voltages (v_1a_ and v_2a_), (**b**) Cross-coherence indicator (Cv_12a_), (**c**) Tripping signal, and (**d**) Auto-coherence indicators (Cv_1a_ and Cv_2a_).

Figure 11a–d illustrate the practical results for case 7. Case 7 is an internal shunt fault (*A13–C13*). Figure 11a presents the two voltages (*v*_*1a*_ and *v*_*2a*_) of the phase *A* stator winding for the machine. Figure 11b depicts the cross-coherence indicator *(Cv*_*12a*_). Figure 11c exhibits the relay tripping signal, and Fig. 11d shows the auto-coherence indicators (*Cv*_*1a*_ and *Cv*_*2a*_). The results of the phase *A* stator winding for the machine during the shunt internal fault (*A13–C13*) are illustrated in Fig. 11. The dissimilarity of the two voltages (*v*_*1a*_ and *v*_*2a*_) causes a differential voltage during the extent of internal fault. In this experiment, the quantities of the coherence indicators (*Cv*_*12a*_*, Cv*_*1a*_ and *Cv*_*2a*_) are roughly + 0.20, + 0.97 and + 0.97, respectively, during the fault span. Therefore, the presence of internal fault activates the algorithm. It is observed that the cross-coherence quantity (*Cv*_*12a*_) is nearly + 0.20, which confirms the internal fault incidence*.* In consequence, the relay operating time is instantaneous in test 7.

### Case 8: internal shunt fault (A14–B14)

**Fig. 12:**
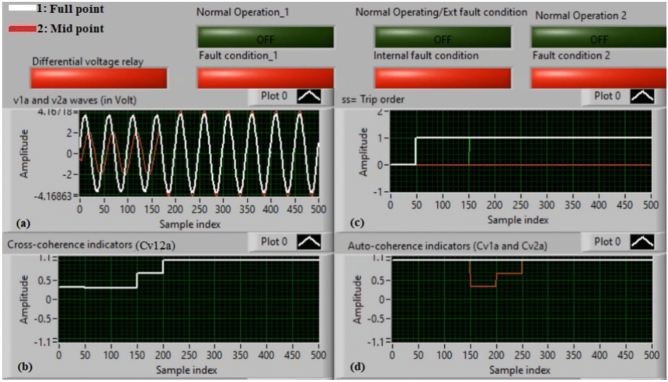
Results for case 8. (**a**) Two measured voltages (v_1a_ and v_2a_), (**b**) Cross-coherence indicator (Cv_12a_), (**c**) Tripping signal, and (**d**) Auto-coherence indicators (Cv_1a_ and Cv_2a_).

Figures 12a–d present the practical results for case 8. Case 8 is an internal shunt fault (*A14–B14*). Figure 12a manifests the two voltages (*v*_*1a*_ and *v*_*2a*_) of the phase *A* stator winding for the machine. Figure 12b shows the cross-coherence indicator *(Cv*_*12a*_). Figure 12c introduces the protection tripping signal, and Fig. 12d depicts the auto-coherence indicators (*Cv*_*1a*_ and *Cv*_*2a*_). The results of the phase *A* stator winding for the machine during the internal fault (*A14–B14*) are presented in Fig. 12. During the period of internal fault, the difference between the two voltages (*v*_*1a*_ and *v*_*2a*_) originates a differential voltage in the relay. In this case, the values of the coherence indicators (*Cv*_*12a*_*, Cv*_*1a*_ and *Cv*_*2a*_) are approximately + 0.18, + 0.97 and + 0.23, respectively, during the fault time. Therefore, the event of internal fault activates the proposed algorithm based on the coherence statistic. It is obvious that the cross-coherence coefficient (*Cv*_*12a*_) is nearly + 0.18, which assures the existence of the internal fault*.* Consequently, the protection tripping time is instantaneous in test 8.

### Case 9: internal shunt fault (A15–C15)

**Fig. 13:**
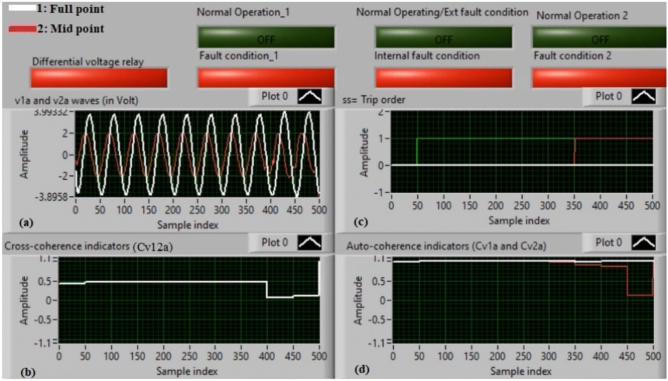
Results for case 9. (**a**) Two measured voltages (v_1a_ and v_2a_), (**b**) Cross-coherence indicator (Cv_12a_), (**c**) Tripping signal, and (**d**) Auto-coherence indicators (Cv_1a_ and Cv_2a_).

Figure 13a–d introduce the obtained results for case 9. Case 9 is an internal shunt fault (*A15–C15*). Figure 13a illustrates the two voltages (*v*_*1a*_ and *v*_*2a*_) of the phase *A* stator winding for the machine. Figure 13b depicts the cross-coherence estimator *(Cv*_*12a*_). Figure 13c illustrates the protection operating announcement, and Fig. 13d presents the auto-coherence estimators (*Cv*_*1a*_ and *Cv*_*2a*_). The outcomes of the phase *A* stator winding for the machine during the internal fault time (*A15–C15*) are shown in Fig. 13. During the time of fault, the difference between the two voltages (*v*_*1a*_ and *v*_*2a*_) causes a differential voltage in the protective relay. In this experiment, the quantities of the coherence estimators (*Cv*_*12a*_*, Cv*_*1a*_ and *Cv*_*2a*_) are roughly + 0.10, + 0.98 and + 0.15, respectively, during the fault time. Thus, the incidence of internal fault activates the developed approach based on the coherence technique. It is evident that the cross-coherence estimator (*Cv*_*12a*_) is about + 0.10, which confirms the presence of the internal fault*.* In consequence, the protection tripping time is instantaneous in test 9.

The experiments on turn-to-turn faults (spanning from case number 10 to case number 13) will be presented below. Quantitative findings for the incidences of turn-to-turn fault are contained in Table 5.

**Table 5:** Quantitative findings for the incidences of turn-to-turn fault.

Case study no	Experimental system state	*v*_*1a*_ (peak value in Volt)	*V*_*2a*_ (peak value in Volt)	*K*_*va*_ = *v*_*2a*_ /* v*_*1a*_	Pickup value	Tripping time (*Tv* (s)_*op*_)
		Select* Kv*_*pu*_ = 0.95*,* and* K*_*s*_ = 0.1				
Case 10	Turn-to-turn fault (A1–A3)—Series fault	3.75	3.40	0.906	*K*_*va*_ < *0.95*	2.060
Case 11	Turn-to-turn fault (A1–A4)—Series fault	3.75	3.10	0.826	*K*_*va*_ < *0.95*	0.666
Case 12	Turn-to-turn fault (A1–A5)—Series fault	3.75	2. 50	0.667	*K*_*va*_ < *0.95*	0.236
Case 13	Turn-to-turn fault (A1–A7)—Series fault	3.75	2.00	0.533	*K*_*va*_ < *0.95*	0.128

### Case 10: turn-to-turn fault (A1–A3)

**Fig. 14:**
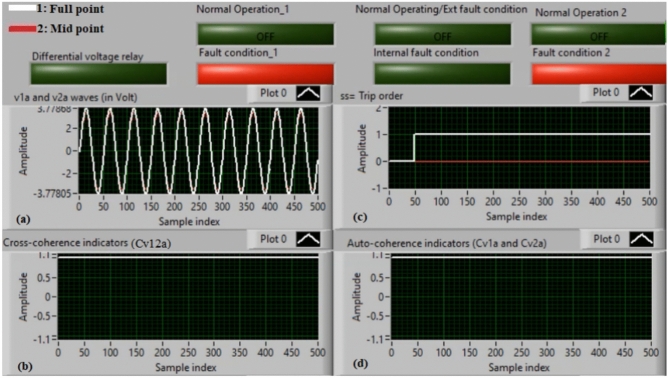
Results for case 10. (**a**) Two measured voltages (v_1a_ and v_2a_), (**b**) Cross-coherence indicator (Cv_12a_), (**c**) Tripping signal, and (**d**) Auto-coherence indicators (Cv_1a_ and Cv_2a_).

Figure 14a–d illustrate the experimental results for case 10. Case 10 is a turn-to-turn fault (*A1–A3*). Figure 14a displays the two voltages (*v*_*1a*_ and *v*_*2a*_) of the phase *A* stator winding for the machine. Figure 14b depicts the cross-coherence estimator *(Cv*_*12a*_). Figure 14c illustrates the protection tripping signal, and Fig. 14d presents the auto-coherence estimators (*Cv*_*1a*_ and *Cv*_*2a*_). The results of the phase *A* stator winding for the machine during the fault period of the turn-to-turn (*A1–A3*) are depicted in Fig. 14. During the fault period, there is a small difference between the two voltages (*v*_*1a*_ and *v*_*2a*_), which causes a differential voltage in the protection. In this experiment, the values of the coherence estimators (*Cv*_*12a*_*, Cv*_*1a*_ and *Cv*_*2a*_) are approximately unity, and the factor *K*_*va*_**≈** 0.906 that is less than *Kv*_*pu*_** = **0.95 during the fault span. These findings affirm the incidence of turn-to-turn fault. Therefore, the fault activates the proposed technique. As a result, the protection tripping time is *Tv*_*op*_**≈** 2.060 s, as given in Table 5.

### Case 11: turn-to-turn fault (A1–A4)

**Fig. 15:**
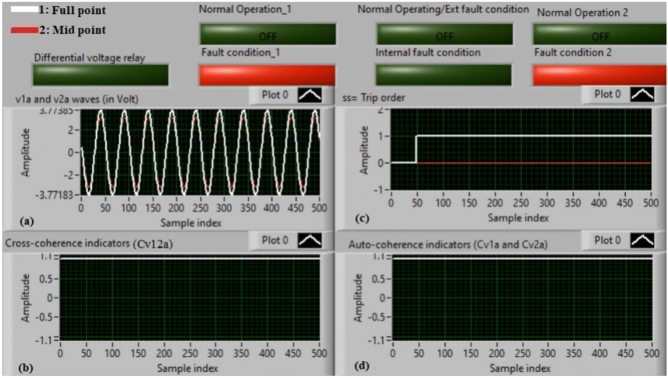
Results for case 11. (**a**) Two measured voltages (v_1a_ and v_2a_), (**b**) Cross-coherence indicator (Cv_12a_), (**c**) Tripping signal, and (**d**) Auto-coherence indicators (Cv_1a_ and Cv_2a_).

Figure 15a–d show the practical results for case 11. Case 11 is a turn-to-turn fault (*A1–A4*). Figure 15a plots the two voltages (*v*_*1a*_ and *v*_*2a*_) of the phase *A* winding for the machine. Figure 15b presents the cross-coherence coefficient *(Cv*_*12a*_). Figure 15c manifests the algorithm tripping signal, and Fig. 15d depicts the auto-coherence coefficients (*Cv*_*1a*_ and *Cv*_*2a*_). The outcomes of the phase *A* stator winding for the machine during the turn-to-turn fault are illustrated in Fig. 15. During the fault time, there is a noticeable difference between the two voltages (*v*_*1a*_ and *v*_*2a*_), which originates a differential voltage in the relay. In this test, the values of the coherence coefficients (*Cv*_*12a*_*, Cv*_*1a*_ and *Cv*_*2a*_) are close to one per unit, and the factor *K*_*va*_**≈** 0.826 that is lower than *Kv*_*pu*_ during the fault interval. These findings assure the event of turn-to-turn fault. As a result, the fault operates the proposed algorithm. Therefore, the protection operating time is *Tv*_*op*_**≈** 0.666 s, as included in Table 5.

### Case 12: turn-to-turn fault (A1–A5)

**Fig. 16:**
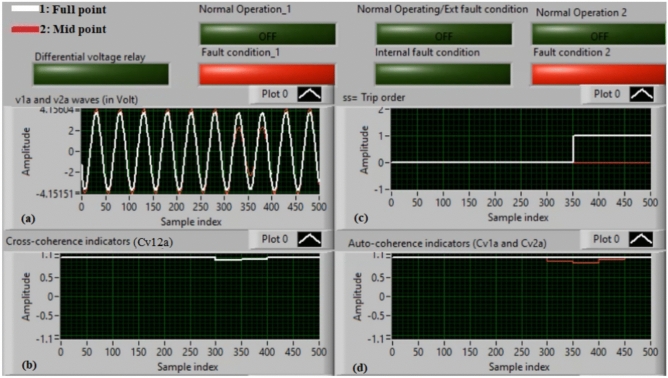
Results for case 12. (**a**) Two measured voltages (v_1a_ and v_2a_), (**b**) Cross-coherence indicator (Cv_12a_), (**c**) Tripping signal, and (**d**) Auto-coherence indicators (Cv_1a_ and Cv_2a_).

Figure 16a–d show the experimental results for case 12. Case 12 is a turn-to-turn fault (*A1–A5*). Figure 16a displays the two voltages (*v*_*1a*_ and *v*_*2a*_) of the phase *A* stator winding for the machine. Figure 16b depicts the cross-coherence estimator *(Cv*_*12a*_). Figure 16c illustrates the protection tripping signal, and Fig. 16d presents the auto-coherence estimators (*Cv*_*1a*_ and *Cv*_*2a*_). The results of the phase *A* stator winding for the machine during the fault period of the turn-to-turn (*A1–A5*) are depicted in Fig. 16. During the fault period, there is a small difference between the two voltages (*v*_*1a*_ and *v*_*2a*_), which causes a differential voltage in the protection. In this experiment, the values of the coherence estimators (*Cv*_*12a*_*, Cv*_*1a*_ and *Cv*_*2a*_) are approximately + 0.85, + 1.0, + 0.80, respectively, and the factor *K*_*va*_**≈** 0.667 that is less than *Kv*_*pu*_** = **0.95 during the fault span. These findings affirm the incidence of turn-to-turn fault. Therefore, the fault activates the proposed technique. As a result, the protection tripping time is *Tv*_*op*_**≈** 0.236 s, as given in Table 5.

### Case 13: turn-to-turn fault (A1–A7)

**Fig. 17:**
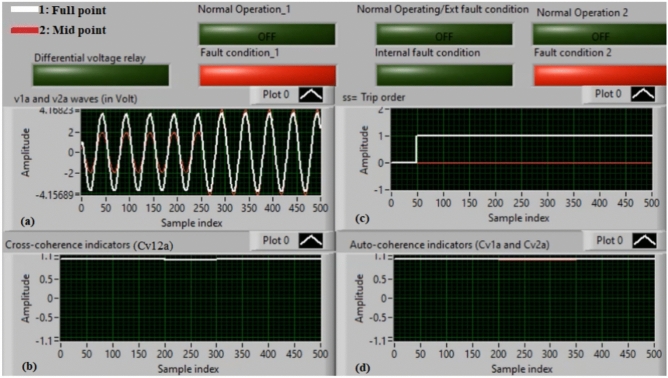
Results for case 13. (**a**) Two measured voltages (v_1a_ and v_2a_), (**b**) Cross-coherence indicator (Cv_12a_), (**c**) Tripping signal, and (**d**) Auto-coherence indicators (Cv_1a_ and Cv_2a_).

Figure 17a–d illustrate the experimental results for case 13. Case 13 is a turn-to-turn fault (*A1–A7*). Figure 17a plots the two voltages (*v*_*1a*_ and *v*_*2a*_) of the phase *A* winding for the machine. Figure 17b presents the cross-coherence coefficient *(Cv*_*12a*_). Figure 17c manifests the algorithm tripping signal, and Fig. 17d depicts the auto-coherence coefficients (*Cv*_*1a*_ and *Cv*_*2a*_). The outcomes of the phase *A* stator winding for the machine during the turn-to-turn fault are illustrated in Fig. 17. During the fault time, there is a noticeable difference between the two voltages (*v*_*1a*_ and *v*_*2a*_), which originates a differential voltage in the relay. In this test, the values of the coherence estimators (*Cv*_*12a*_*, Cv*_*1a*_ and *Cv*_*2a*_) are nearly + 0.90, + 1.0, + 0.95, respectively, and the factor *K*_*va*_**≈** 0.533 that is lower than *Kv*_*pu*_ during the fault interval. These findings assure the event of turn-to-turn fault. As a result, the fault operates the proposed algorithm. Therefore, the protection operating time is *Tv*_*op*_**≈** 0.128 s, as included in Table 5.

The experimental tests on external shunt faults, ranging from case number 14 to case number 16), will be illustrated as follows:

### Case 14: external shunt fault (A5–B5)

**Fig. 18:**
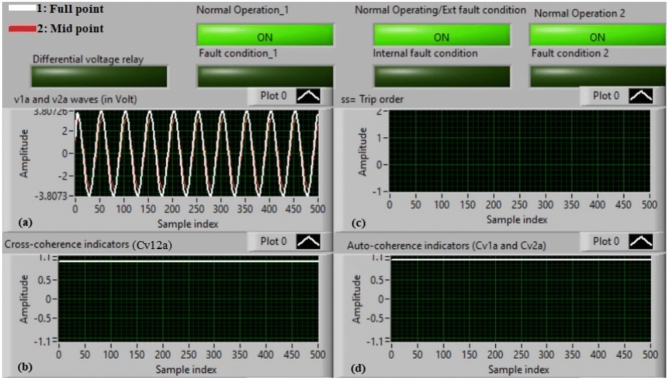
Results for case 14. (**a**) Two measured voltages (v_1a_ and v_2a_), (**b**) Cross-coherence indicator (Cv_12a_), (**c**) Blocking signal, and (**d**) Auto-coherence indicators (Cv_1a_ and Cv_2a_).

Figure 18a–d illustrate the practical findings for case 14. Case 14 is an external shunt fault (*A5–B5*). Figure 18a introduces the two voltages (*v*_*1a*_ and *v*_*2a*_) of the phase *A* stator winding for the AC machine. Figure 18b presents the cross-coherence indicator (*Cv*_*12a*_). Figure 18c declares the blocking signal, which its value is zero during the full display time, and Fig. 18d offers the two auto-coherence indicators (*Cv*_*1a*_ and *Cv*_*2a*_). As depicted in Fig. 18, the experimental results illustrate that the state of phase *A* stator winding is healthy. The reason is that the two voltages signals (*v*_*1a*_ and *v*_*2a*_) are nearly corresponding, and there is no difference between the two voltages. In this test, the quantified results are similar to that recorded in test 1. The values of the coherence indicators (*Cv*_*12a*_*, Cv*_*1a*_ and *Cv*_*2a*_) are nearly 0.95. Consequently, the proposed differential voltage protection based on the coherence technique is inactive due to the external fault. As a result, the relay tripping time is infinity in this experiment.

### Case 15: external shunt fault (A6–B6)

**Fig. 19:**
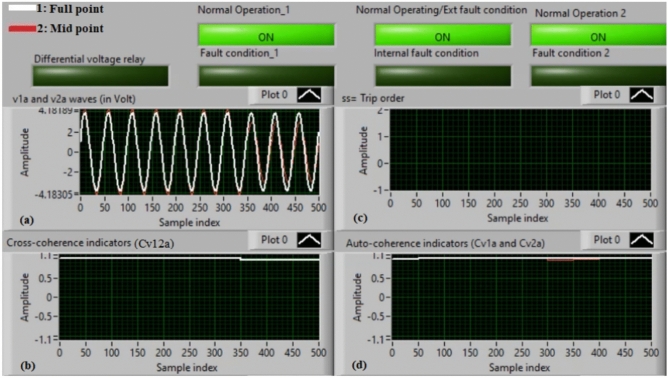
Results for case 15. (**a**) Two measured voltages (v_1a_ and v_2a_), (**b**) Cross-coherence indicator (Cv_12a_), (**c**) Blocking signal, and (**d**) Auto-coherence indicators (Cv_1a_ and Cv_2a_).

Figure 19a–d illustrate the experimental results for case 15. Case 15 is an external shunt fault (*A6–B6*). Figure 19a offers the two voltages (*v*_*1a*_ and *v*_*2a*_) of the phase *A* stator winding for the induction machine. Figure 19b illustrates the cross-coherence indicator (*Cv*_*12a*_). Figure 19c declares the blocking signal, which its value is zero during the full display time, Fig. 19d introduces the two auto-coherence indicators (*Cv*_*1a*_ and *Cv*_*2a*_). As shown in Fig. 19, the experimental results affirm that the state of phase *A* stator winding is normal and healthy. The cause is that the two voltages signals (*v*_*1a*_ and *v*_*2a*_) are roughly identical, and there is a small difference between the two voltages. In this experiment, the obtained results are similar to that recorded in experiment 1. The values of the cross-coherence indicator *(Cv*_*12a*_) are about 0.96, and the values of the auto-coherence indicators *(Cv*_*1a*_ and *Cv*_*2a*_) are about one. As a result, the differential voltage protection based on the coherence algorithm is blocking; leading to the protection tripping time is infinity.

### Case 16: external shunt fault (A7–B7)

**Fig. 20:**
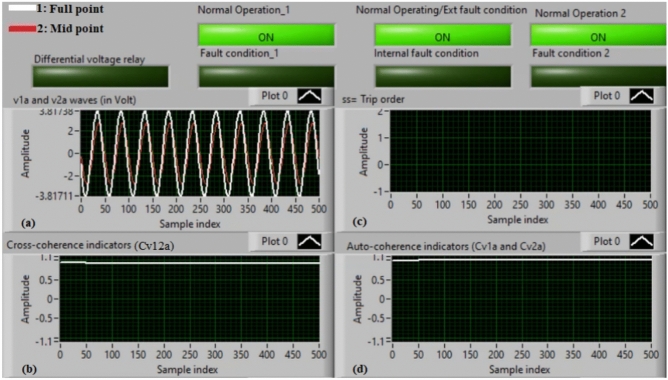
Results for case 16. (**a**) Two measured voltages (v_1a_ and v_2a_), (**b**) Cross-coherence indicator (Cv_12a_), (**c**) Blocking signal, and (**d**) Auto-coherence indicators (Cv_1a_ and Cv_2a_).

Figure 20a–d present the experimental results for case 16. Case 16 is an external shunt fault (*A7–B7*). Figure 20a depicts the two voltages (*v*_*1a*_ and *v*_*2a*_) of the phase *A* stator winding for the machine. Figure 20b introduces the cross-coherence indicator (*Cv*_*12a*_). Figure 20c shows the blocking signal, which its value is zero during the full display time, Fig. 20d illustrates the two auto-coherence indicators (*Cv*_*1a*_ and *Cv*_*2a*_). As shown in Fig. 20, the experimental results affirm that the state of phase *A* stator winding is normal and healthy. This is because the two voltages signals (*v*_*1a*_ and *v*_*2a*_) are roughly identical, and there is a small difference between the two voltages. In this test, the results are similar to that recorded in test 1. The values of the cross-coherence indicator *(Cv*_*12a*_) are about 0.92, and the values of the auto-coherence indicators *(Cv*_*1a*_ and *Cv*_*2a*_) are about one. As a result, the differential voltage protection based on the coherence algorithm is restraining; resulting in the protection operating time is infinity.

The obtained results manifest that the coherence-based differential voltage protection for the induction machine stator windings is able to:

1. Discriminate between the healthy and faulty states (healthy state is presented in case number 1, and faulty states are illustrated in cases 2–9),
2. Distinguish between the states of internal and external faults with regard to the protection zone of the machine (internal faults are depicted in cases 2–9, and external faults are shown in cases 14–16),
3. Detect the turn-to-turn faults, and estimate the tripping times in these faults (as shown in cases 10–13),
4. Develop a novel tripping-characteristic curve based on the coherence indicators calculated for machine voltage waves (as presented in Fig. 1), which is created to differentiate between turn-to-turn, external and internal shunt faults,
5. Assess the unbalance degree using the cross-coherence indicators estimated between each two phases of the three-phase voltages, and
6. Classify the shunt faults located inside the machine protection region (as illustrated in Fig. 3a,b),

For the sixteen case studies, a comparison of power quality parameters of the two measured voltages (*v*_*1a*_ and *v*_*2a*_) is presented in Table 6.

**Table 6:** Comparison of power quality parameters of the two measured voltages (*v*_*1a*_ and *v*_*2a*_).

Case study no	Experimental system state	Power quality parameters of the two measured voltages (*v*_*1a*_ and *v*_*2a*_)				
		Magnitude	Frequency	Phase shift	Sine wave	Polarity
Case 1	Normal operation (Phase A)	**√**	**√**	**√**	**√**	**√**
Case 2	Internal shunt fault (A11–B11)	**x**	**x**	**x**	**√**	**√**
Case 3	Internal shunt fault (A11–C11)	**x**	**x**	**x**	**√**	**√**
Case 4	Internal shunt fault (A12–B12)	**x**	**x**	**x**	**√**	**√**
Case 5	Internal shunt fault (A12–C12)	**x**	**x**	**x**	**√**	**√**
Case 6	Internal shunt fault (A13–B13)	**x**	**x**	**x**	**√**	**√**
Case 7	Internal shunt fault (A13–C13)	**x**	**x**	**x**	**√**	**√**
Case 8	Internal shunt fault (A14–B14)	**x**	**x**	**x**	**√**	**√**
Case 9	Internal shunt fault (A15–C15)	**x**	**x**	**x**	**√**	**√**
Case 10	Turn-to-turn fault (A1–A3)—Series fault	**x**	**√**	**√**	**√**	**√**
Case 11	Turn-to-turn fault (A1–A4)—Series fault	**x**	**√**	**√**	**√**	**√**
Case 12	Turn-to-turn fault (A1–A5)—Series fault	**x**	**√**	**√**	**√**	**√**
Case 13	Turn-to-turn fault (A1–A7)—Series fault	**x**	**√**	**√**	**√**	**√**
Case 14	External shunt fault (A5–B5)	**√**	**√**	**√**	**√**	**√**
Case 15	External shunt fault (A6–B6)	**√**	**√**	**√**	**√**	**√**
Case 16	External shunt fault (A7–B7)	**√**	**√**	**√**	**√**	**√**

**√** means similar, and **x** means dissimilar for the two voltages (*v*_*1a*_ and *v*_*2a*_).

## Protection characteristics

### Protection properties assessment

The characteristics of the present method have been assessed over a period of four months. During this span, the total number of trips in this project was 970.0, out of which 11.0 were incorrect. The total number of instances where the protection method failed to trip was 19.0. The protection was restrained 90 times without any problems when the system was operating normally. In Table 7, the quantitative findings regarding the algorithm’s properties under the influence of diverse fault types and voltage transformer errors are presented^39,40^.

**Table 7:** The quantitative findings of the algorithm’s properties.

Experimental system state	Number of instances	Malfunction times
The total number of trips	970	11
The total number of normal operations	90	0
The quantitative findings of the algorithm’s properties	C_1_ = The total number of tests = 1060	Malfunction times = 11
	C_2_ = The total number of trips	= 970
	C_3_ = The number of correct trips	= 959
	C_4_ = The number of tripping failures	= 19
	C_5_ = The number of desirable trips	= 959 + 19 = 978
	C_6_ = The number of incorrect trips	= 970– 959 = 11
	C_7_ = C_5_ + C_6_	= 978 + 11 = 989
	$D = \frac{C3 }{{C5}} \times 100\%$ ^39,40^	= $\frac{959}{{978}}$ $\times$ 100 = $98.1 \%$
	$S = \frac{C3}{{C2}} \times 100 \%$ ^39,40^	= $\frac{959}{{970}} \times$ 100 = $98.87 \%$
	$R = \frac{C3}{{ C7}} \times 100\%$ ^39,40^	= $\frac{959}{{989}} \times$ 100 = $96.97 \%$
	$A = \frac{C1 - C4 - C6}{{C1}} \times 100 \%$ ^39,40^	= $\frac{1060-19-11 }{1060} \times$ 100 = 97.17%

Remark: D = Dependability, S = Security, R = Reliability, and A = Accuracy

### Algorithm merits

The benefits of the coherence indicators are summarized below.

1. The data window area can be utilized to control both the protection speed and the fault detection time. The smaller the data window, the lower the protection accuracy and the faster the protection speed,
2. The association and synchrony degrees of the three-phase voltage signals can be identified and evaluated simultaneously using the coherence measure. Therefore, it is convenient to measure the severity of the unbalanced voltages,
3. A tripping curve is designed based on the coherence indicators for the voltage signals. This curve can be harnessed to trip the machine circuit breakers when the incidences of the fault or imbalance occur to avoid the system harm; while, it prevents the protection operation in the situations of voltage balance and normal operation for the machine,
4. The protection can readily set the convenient coherence threshold values to differentiate between the faulty and healthy states, as well as distinguish between the internal and external shunt faults,
5. A balance between the protection speed and accuracy can be considered because high-speed protection systems tend to be less accurate,
6. The cross-coherence indicators combine the influences of zero and negative sequence components to determine and measure the voltage asymmetry. Thereby, the coherence is considered a suitable instrument to assess the di-symmetry coefficients,
7. The protection sensitivity can be set using the data window distance and the coherence setting divergences,
8. In practice, the coherence threshold values can be adjusted by observing the alarm flag and protection response signal during normal operation, as well as the acceptable unbalance of the machine waveforms on the LABVIEW platform. As the coherence settings are closer to + 1.0, as the relay accuracy is lower,
9. To increase the computational efficiency, the measurement errors should be avoided using the following:
  - A suitable data set should be chosen to eliminate the voltage measurement errors and acceptable ripples,
  - Differential mode should be selected for the analog-to-digital converter,
  - It is important to found a proper grounding in the experimental system,
  - Anti-aliasing criterion should be used in digital signals processing,
  - The voltage transformers should be from the same manufacturer, and have the same accuracy class and VTR.
10. To investigate the algorithm reliability in larger-scale machines, several factors should be followed:
  - The algorithm uses two voltage waves (as analog inputs) for each phase to monitor changes in these voltages at once,
  - The experimental system should be tested under extensive and diverse fault conditions, such as turn-to-turn and shunt faults,
  - A new configuration of tripping characteristic has been set up, which can be harnessed to make the algorithm active when a fault or imbalance event happens to prevent any machine damage. Whereas, the algorithm is inactive when the machine is normal and its output voltages are balanced,
  - Accurate design, proper components, and correct installation and testing are necessary for the whole protection system to guarantee its reliability. This involves the reliability of diverse elements, such as analog input circuits, digital relays, and the digital output circuits.
  - Duplicating the algorithms or two analog input signals to the protection system can attain the protection reliability,
  - To verify the efficient and effective protection algorithm, a variety of tests should be conducted, including but not limited to:
    (I) *Simulation testing* The machine behaviors under diverse fault scenarios have been simulated utilizing the ATP program, and the MATLAB program has been exploited to process the protection algorithm. The simulation testing can verify the coherence settings and logic of the algorithm. However, the simulation examination faces certain obstacles, like the model accuracy, the difficulty of simulated complex faults, and the running time required for the simulation.
    (II) *Primary injection testing* The protection algorithm has been verified through primary injection testing, which has been conducted by injecting voltage signals into the protection system and measuring the protection response. This test necessitates eventually a personal computer, an analog-to-digital converter, and LABVIEW software. The primary injection testing can confirm the functionality, sensitivity, and accuracy of the protection system under actual situations, as well as its performance. Nonetheless, the primary injection testing faces several obstacles, such as the safety of the participants and the availability of large-scale machines.
11. The coherence settings can be exploited to control the restraining and operating regions contained within the tripping characteristic curve of the protection. The subsequent points demonstrate their impact on the protection characteristics:
  - An increase in both the data set area and the blocking region located within the tripping characteristics can boost the protection security,
  - Reducing both the data set area and the blocking region located within the tripping characteristics can increase the protection sensitivity.
  - The application of the data set principle to measure the coherence indicators filters the input waves and delays the protection operation. This verifies that the protection is stable, leading the system to continue to be stable under the following conditions: VT measurement errors, acceptable harmonics, and transient faults,
  - The algorithm is highly reliable because of its immunity to various fault types, fault onset times, fault time intervals, and fault locations. It can also identify the vast majority of fault types,
  - The technique satisfies the protection’s reliability and stability. The protection’s reliability is mainly measured by the absence of failures in the relay operation, while the protection’s stability is determined by the protection’s efficiency for recognizing system operating conditions,
  - A compromise in the protection settings is essential to coordinate between dependability and stability, accuracy and speed, and sensitivity and security. Therefore, the values of the coherence settings selected are 0.05, and the data set area is taken as a single cycle in the proposal.

### Comparative assessment of the suggested scheme

Table 8 presents a crucial comparison between the suggested coherence-based differential voltage protection and other recent approaches in terms of performance metrics (such as main concept, functional role, configuration of tripping curve, protection accuracy, computation time, protection settings, and asymmetry ratios).

**Table 8:** A comparison between the suggested coherence-based differential voltage protection and other recent approaches.

Article	Demerits	Merits of coherence-based differential voltage protection
^36,38^	1- Most protection techniques used the RMS currents for estimating differential and biasing currents in the differential current relay^36,38^ 2- The pickup differential current is dependent on the current transformer (CT) measurement error, and the low internal fault currents^36,38^ 3- The differential current relay depends on the proportion of the differential current quantity to the biasing current quantity to figure out the internal shunt faults^36,38^ 4- Many protection methods necessitate offline studies, while others are implemented in real-time^36,38^ 5- The operating characteristics of the differential current protection systems have an open curve and their boundary is not definite^36,38^ 6- The characteristic settings modification for the differential current protection is related to the machine parameters’ data and the protection properties needed, as well as the specifications of the instrument transformer^36,38^ 7- The tripping characteristic settings of the differential current protection should be neatly estimated to prevent the protection from malfunctioning. The pick-up differential current of the traditional differential current protection depends on the equipment specifications. Moreover, the characteristic slope in the differential current protection should be minutely selected^36,38^ 8- Several techniques of fault diagnosis for electrical machine stator windings used complicated offline estimates to equip protection settings 9- The characteristic of the protection integration is not obtainable in multiple methods, thereby reducing the reliability of the protection system^36,38^	1- The method is dependent on nine coherence indicators that are computed for six voltages taken at the midpoints and full terminals of the three-phase stator winding of the machine 2- The pickup values are contingent upon the prescribed coherence settings, which are restricted to a limited area of the relay tripping characteristic 3- The protection operation depends on the three cross-coherence indicators to identify and classify the internal shunt faults, and select the faulty phase(s), while, the six auto-coherence indicators are used to make assure that the machine is either faulty or healthy 4- The approach can figure out the faults online 5- The protection tripping curve is bounded and quadratic (where, it relies on the coherence indicators that range from 0.0 to + 1.0) 6- The turn-to-turn, external and internal faults can be distinguished by utilizing the blocking and tripping areas existing within the designed tripping curve 7- The protection attributes can be modified by the predetermined coherence settings and the data set size 8- Coherence settings are used to modify the tripping and restraining zones situated within the coherence-based tripping characteristic without requiring any mathematical operation 9- The advanced approach can be incorporated with other digital systems (including numerical protective relays, event recorders, and fault recorders) to increase the reliability and integration of the protection system, 10- The proposed algorithm combines several protection functions, such as phase differential voltage, and unbalanced voltage protections
^24,25^	1- The methods do not use backup protection techniques^24,25^	1- The present method employs three cross-coherence indicators and six auto-coherence indicators, which boosts the protection redundancy of the three-phase machine stator windings
^30^	1- Its practical application is extremely sophisticated^30^	1- It can be described as a simple protection technique. Therefore, it can be easily used in practice
^35,37^	1- In the incidence of turn-to-turn or symmetrical faults, there are several fault diagnosis approaches that are unreliable, undependable, unsecure, and inaccurate^35,37^ 2- The operating time of the differential and restricted earth fault relays is dependent on the RMS values, which take a duration of one cycle of the nominal power frequency^35,37^ 3- The longitudinal type of differential current protection can not specify the onset of turn-to-turn fault because this type of fault is unrealizable, although it is harmful ^35^, 4- Multiple approaches are unable to identify the faults occurring from turn-to-turn in single-winding machines 5- The differential current protection of the transverse type is capable of detecting the turn-to-turn faults in electrical machines with dual windings per phase	1- It substantiates high ratios of protection algorithm reliability, dependability, security, and accuracy 2- The prescribed quantity of the data set can be used to regulate the computation time and the fault detection time of the algorithm. It can be determined within a single cycle of the fundamental power frequency 3- It is capable of defining the faults that occur from turn to turn in the machine windings 4- It is applicable to find out turn-to-turn faults, whether machine is single-winding or dual-windings per phase 5- This approach can be used to protect single or multi-winding machines
^39,40^	1- In reference^39^, the technique employed the arithmetic expressions for the alienation coefficients obtained using the coherence coefficients calculated for the three-phase voltages and currents of the synchronous generator. Accordingly, the computational time of the alienation-based protection algorithm exceeds that of the coherence-based protection algorithm 2- In reference^39^, cross-alienation coefficients for the measured three-phase voltages and currents of the synchronous generator were used to value the asymmetry rates 3- In reference^39,40^, the technique lacked the capability to: (a) Find out the faults that occur due to short-circuit turns, (b) Discriminate between shunt faults inside and outside the equipment protection region, and (c) Categorize the shunt faults located inside the protection zone of the machine 4- Several methods perform certain protection functions, and protect against definite types of faults^39,40^ 5- Several methods used the three-phase currents at the two ends of the equipment’s stator windings^35,37^, and others used the three-phase voltages and currents at the load terminal of the equipment^39,40^	1- The approach has applied direct mathematical expressions for the coherence indicators estimated for only the machine three-phase voltages. Accordingly, the computational time of the coherence-based differential voltage protection is less than that of the alienation-based protection. This leads to a faster fault detection time 2- The cross-coherence indicators have immediately been used to measure the asymmetry rates for the voltage measurements of the machine 3- The methodology has the ability to: (a) Determine the inter-turn faults, (b) Distinguish between shunt faults inside and outside the equipment protection region, (c) Classify the shunt faults located within the machine protection area, (d) Examine the asymmetry of three-phase voltages, and (e) Evaluate the degree of disturbance intensity in the three-phase voltages 4- The voltages, which are measured at the midpoint and complete terminal of each stator winding of the machine, are acquired to estimate the three coherence indicators for each phase. Consequently, the transmission and processing of data are swift
^47^	1- In reference^47^, harmonic components analysis used a greater sampling frequency. The selected sampling frequency of 78.125 kHz (i.e., 256 samples per cycle) would result in a delay in computational time	1- The technique has used a sampling frequency of 2.5 kHz (i.e., 50 samples per cycle). Consequently, it works faster than the technique in^47^ 2- In the present methodology, the asymmetry assessment process is accomplished after passing a single cycle, provided that the predetermined data set is specified as a single cycle. Furthermore, the selected data window controls the algorithm’s operational time
^45^	1- In reference^45^, the voltage asymmetry model stated by the IEC was used to estimate the percentages of negative-sequence relative to positive-sequence components. The approach disregarded the zero-sequence components. As a result, the approach in^45^ has a lower accuracy than that of the present method 2- In reference^45^, the Root Mean Square (RMS) quantities of the three-phase voltages were employed to estimate the asymmetry coefficient 3- In reference^45^, the approach used only the three-phase voltages of the power supply	1- To determine and estimate the asymmetry level, the cross-coherence indicators (which are computed between each two phase voltages) consider the influence of the combination for both zero-sequence and negative-sequence components. Additionally, the coherence indicators can detect changes in any power quality parameter. Thus, the coherence indicator is a commendable instrument for evaluating the asymmetry factors 2- The estimation of asymmetry relies on the coherence indicators calculated for voltage measurements per data set. The data set area can be altered according to the prevailing operations of the power system and the protection requirements 3- The developed method has exploited the six voltage waves measured at the midpoints and complete terminals for the three-phase stator windings of the machine
^46^	1- In reference^46^, several mathematical formulas were applied, resulting in various setting values that were set to accommodate diverse power quality phenomena in power systems 2- In reference^46^, the protection scheme took more time to derive the compound index employed to assess power quality phenomena	1- The approach values the asymmetry ratios using the coherence indicators, and the asymmetry evaluation process takes about a single cycle of the fundamental power frequency

## Algorithm assumptions

To stratify this approach in large-scale or more complicated systems, several assumptions must be considered, as listed below.

1. Certain common requirements should be satisfied for the voltage transformers and their measurements, as follows:
  - Voltage transformers are the same transformer type used in three-phase circuits,
  - Voltage transformers have the same accuracy class,
  - Voltage transformers have the same ratio of turns, and are the same size, and.
  - Voltage measurements should be perfectly synchronized.
2. The coherence-based protection algorithm requires the following:
  - A large sample quantity can yield statistically significant findings, even if the coefficient of coherence is small. Thus, a significant sample size should be considered, and.
  - The coherence estimator lacks sensitivity to the transformation and scale, so the algorithm has not responded to several fault cases.
3. To relieve the incidence of unsound tripping of the algorithm, arising from power quality troubles, certain provisions should be taken, including the following:
  - The data set and coherence settings should be reasonable,
  - The analog-to-digital converter should be set to work in differential mode,
  - It is imperative to establish a proper grounding system within the practical system,
  - Proper instrument transformers with the same type, rating, accuracy class, and ratio of turns should be considered,
  - Digital sensors, meters, and LABVIEW software, should be used to take measurements of electrical signals and record continuously the parameters of the power quality,
  - The antialiasing principle and the Nyquist sampling theorem should be applied.

## Contributions

A list of contributions from the authors is presented below.

1. A new coherence-based method of differential voltage protection for induction machine stator windings is proposed. Only three-phase voltages are acquired at the two terminals of the stator windings of the AC machines to process the differential protection,
2. A new connection circuit for the differential voltage relay has been established between the mid and full points of each winding, which can be used to protect the entire windings of power transformers, synchronous machines, and induction machines. This design can protect single-phase and three-phase machines, as well as the equipment with single or dual windings per phase,
3. To discriminate between the instances of turn-to-turn faults, and external and internal faults, a novel design of quadratic operating characteristic curves based on the coherence instrument is presented.
4. The shunt faults existing within the AC machine protection zone can be immediately identified and classified using the cross-coherence indicators. Furthermore, a novel mathematical model can be used to quantify the relay operating time in the instance of turn-to-turn faults, and.
5. The intensity degree of imbalance and fault for the three-phase voltages can be specified and evaluated utilizing the cross-coherence and auto-coherence indicators, respectively.

## Conclusions

A coherence-based differential voltage protection for the machine stator windings protection has been proposed. To process the proposed algorithm, three-phase voltages have been measured at the midpoint and complete end of each stator winding of the machine. The voltage waveforms have been measured with the DAC, and the algorithm has been run in LABVIEW software. The methodology has been tested on a three-phase induction machine that contains 20 taps for each phase stator winding. This configuration is designed to install voltage transducers at the center points and the full terminals of the three-phase stator windings, and to facilitate thorough examinations to assess the algorithm’s effectiveness and efficiency. Various fault incidences have been investigated, such as winding-to-neutral, winding-to-winding, and turn-to-turn faults. The testing results have shown that the algorithm security and dependability rates are greater than 98%, and the algorithm reliability and accuracy rates are about 97.0%. Actually, the differential voltage protection has the capability to detect a fault occurrence, define turn-to-turn faults, and distinguish shunt faults inside and outside the machine zone. It can also classify ten types of shunt faults in the machine protection zone. The quantitative findings have demonstrated that the response time for detecting the internal shunt fault has been achieved within 20 ms, indicating an immediate response for the relay. Whereas, in the event of the turn-to-turn fault, the operating time has been contingent upon its severity level, which has been estimated using a novel mathematical expression. Besides, a new form of relay operating curves, based on the coherence indicators, has been founded to contrast turn-to-turn faults, external and internal faults. Moreover, the approach can assess the ferocity grade of the three-phase voltages imbalance and troubles, and it can protect single-phase and three-phase windings of various AC machines, as well as power transformers. It can be applied to protect the AC machines with single or dual windings per phase as well. Furthermore, the protection sensitivity, security, dependability, and fault detection time can be controlled by modifying coherence settings and data window size.

## Supplementary Information

Supplementary Information.

## Data Availability

All data generated or analysed during this study are included in this published article [and its supplementary information files].
