# Supplementary material for: Experimental evaluation of differential voltage protection scheme based on a coherence function applied to AC machine stator windings
Source: Sci Rep. 2025 Apr 11;15:12399. doi: 10.1038/s41598-025-93210-2 (PMC11992023; doi:10.1038/s41598-025-93210-2)
Supplement: Supplementary file 1 — Supplementary Information. [file 41598_2025_93210_MOESM1_ESM.pdf]

Nomenclatures:

Symbols	Abbreviations	Symbols	Abbreviations
VT	Voltage Transformer	$v_{1b}(n-N_c)$	The measurement of the voltage (v_{1b}) at the instant time ($n-N_c$),
VTR	Voltage Transformer Ratio	$v_{2b}(n-N_c)$	The measurement of the voltage (v_{2b}) at the instant time ($n-N_c$),
CB	Circuit Breaker	$v_{1c}(n-N_c)$	The measurement of the voltage (v_{1c}) at the instant time ($n-N_c$),
DAC	Data Acquisition Card	$v_{2c}(n-N_c)$	The measurement of the voltage (v_{2c}) at the instant time ($n-N_c$),
SLNF	Single-Line-to-Neutral Fault	*TTTFs*	Turn-to-Turn Faults
DLF	Double Line Fault	N_c	The number of measurements per each cycle of the nominal power frequency for each voltage signal,
DLNF	Double Line-to-Neutral Fault	N_s	The number of measurements per each data set of the voltage signal,
3LNF	Three Line-to-Neutral Fault	Cv_{12x}	The cross-coherence indicator computed between each two conforming data sets for the two voltages ($v_{1x}(n)$ and $v_{2x}(n)$) measured for the '*X*' phase of the induction machine stator winding,
$v_{1a}(n)$, $v_{1b}(n)$ and $v_{1c}(n)$:	At the instant *n*, the voltage values of the *a*, *b* and *c* phases, respectively, are acquired at the terminals of the full stator windings for the three-phase induction machine,	Cv_{1x}	The auto-coherence indicator estimated between each two successive data sets differing in time by one cycle for the voltage $v_{1x}(n)$ measured at the full stator winding of the 'X' phase for the induction machine,
$v_{2a}(n)$, $v_{2b}(n)$ and $v_{2c}(n)$:	At the instant *n*, the voltage values of the *a*, *b* and *c* phases, respectively, are acquired at the mid-points of the stator windings for the three-phase induction machine,	Cv_{2x}	The auto-coherence indicator calculated between each two successive data sets differing in time by one cycle for the voltage $v_{2x}(n)$ measured at the mid-point of the stator winding of the 'X' phase for the induction machine,
$v_{1a}(n-N_c)$	The measurement of the voltage (v_{1a}) at the instant time ($n-N_c$),	*IM*	Induction motor,
$v_{2a}(n-N_c)$	The measurement of the voltage (v_{2a}) at the instant time ($n-N_c$),	Δx_1, and Δx_2	The divergences of coherence setting,
X	The subscript *X* is the designated phase *A*, *B*, or *C*.	Δx_1	The prescribed setting divergence of the cross-coherence indicator (Cv_{12x}),
X and *Y*	The subscript *X* or *Y* is the designated phase *A*, *B*, or *C*, but they are two different phases of the three phases,	Δx_2	The prescribed setting divergence of the auto-coherence indicator (Cv_{1x} or Cv_{2x}),
Cv_{1xy}	The cross-coherence indicator estimated between each two conforming data sets for the voltages ($v_{1x}(n)$ and $v_{1y}(n)$) taken for the two phases '*X* and *Y*' of the induction machine stator windings,	Cv_{2xy}	The cross-coherence indicator calculated between each two conforming data sets for the voltages ($v_{2x}(n)$ and $v_{2y}(n)$) taken for the two phases '*X* and *Y*' of the induction machine stator windings,
VTX_2	A voltage transformer with turns' ratio of 220/6 installed at the midpoint of the phase winding (*X*),	VTX_1	A voltage transformer with turns' ratio of 220/3 built at the complete terminal of the phase winding (*X*),

Tv_{op}	The quantified tripping time of the algorithm (in Sec),	K_s	The time multiplier (in this algorithm, K_s is selected 0.1),
Kv_{pu}	The predetermined pickup ratio of the relay (in this algorithm, Kv_{pu} is selected 0.95),	Kv_x	The coefficient computed using the peaks' ratio of the two voltage signals (v_{1x} and v_{2x}) for phase X,
v_{1xpeak}	The measured peak value of the voltage signal $v_{1x}(n)$,	v_{2xpeak}	The measured peak value of the voltage signal $v_{2x}(n)$.

Appendix 1: The parameters' specifications of the power model elements

The specifications of the power system elements	Numerical value
**Three-phase power supply:**	
Rated line voltage	*380 V*
Rated frequency	*50 Hz*
**Three-phase induction motor (under test):**	
Rated power	*2.9 kW (Star connection)*
Rated line voltage	*400 V*
Nominal frequency	*50 Hz*
Rated line current	*6.3 A*
Rated speed	*1415 rpm*
No. of taps per each winding	*20 Tapes*
No. of turns per each tap	*5 Turns*
Turn dimension	*0.8* mm^2
**Current transformers (CTs):**	
Current Transformer turns' Ratio (CTR)	*200/5*
Frequency	*47…50…63 Hz*
CT accuracy class	*1.0*
Rated burden	*2.5 VA*
CT burden	*1 Ω*
**Voltage Transformers (VTs)**	
Voltage Transformer turns' Ratio (VTR)	*220 / 6/ 3*
VT accuracy class	*0.5*
Nominal frequency	*50/60 Hz*

Rated burden	*25 VA*
**Miniature Circuit Breaker (MCB1)**<br><br>Phase type<br><br>Rated current<br><br>Rated voltage	 <br>Three phase<br><br>63 A<br><br>400 V

Appendix 2: Input quantities of the proposed protection algorithm

Quantity designation	Quantity description	Value
$v_{1a}(n)$, $v_{1b}(n)$ **and** $v_{1c}(n)$	At the instant *n*, the voltage values of the *a, b* and *c* phases, respectively, are acquired at the terminals of the full stator windings for the induction machine,	The voltage measurements are acquired on-line
$v_{2a}(n)$, $v_{2b}(n)$ **and** $v_{2c}(n)$	At the instant *n*, the voltage values of the *a, b* and *c* phases, respectively, are acquired at the mid-points of the stator windings for the induction machine,	
F_c	The fundamental power frequency,	50 *Hz*
T_c	The cycle time period,	20 milliseconds
F_{sp}	The sampling frequency,	2.5 *kHz*
T_{sp}	The sampling time,	0.4 milliseconds
N_c	The samples size per each one cycle, $N_c = T_c / T_{sp}$ or $N_c = F_{sp} / F_c$	50 samples/cycle
N_s	The samples size per each data set, $N_s = N_c$	50 samples/data set
T_{ds}	The full display time,	10 cycles
Δx_1	The presetting deviation for the cross-coherence indicators: (Cv_{12a}, Cv_{12b} and Cv_{12c}),	+0.05
Δx_2	The presetting deviation for the auto-coherence indicators: (Cv_{1a}, Cv_{1b}, Cv_{1c}, Cv_{2a}, Cv_{2b} and Cv_{2c}),	+0.05
